# Modulation of microbiota by traditional Chinese medicine in chronic inflammatory airway disease: a systematic review and meta-analysis of animal studies

**DOI:** 10.3389/fimmu.2026.1904457

**Published:** 2026-09-30

**Authors:** Junting Sai, Haixia Li, Liu Chun, Weike Li, Dongmin Song, Mingyang Yi, Zhiwan Wang

**Affiliations:** 1 National Regional TCM (Pulmonary Disease) Diagnostic and Treatment Center, The First Affiliated Hospital of Henan University of Chinese Medicine, Zhengzhou, China; 2 Department of Respiratory Medicine, The First Affiliated Hospital of Henan University of Chinese Medicine, Zhengzhou, China; 3 The First Clinical Medical School, Henan University of Chinese Medicine, Zhengzhou, China; 4 Henan University of Chinese Medicine, Zhengzhou, China

**Keywords:** chronic obstructive pulmonary disease, asthma, microbiome, alpha diversity, beta diversity, relative abundance, meta-analysis

## Abstract

**Background:**

A growing number of studies have indicated that traditional Chinese medicine (TCM) can regulate microbiota in chronic inflammatory airway disease (CIAD). Nevertheless, no systematic review or integrated analysis has summarized relevant evidence to date, limiting the clinical application of TCM in this field.

**Methods:**

This study followed the PRISMA reporting guidelines. We systematically searched PubMed, EMBASE, the Cochrane Library and Web of Science for animal studies published from January 1, 2006 to January 5, 2026 exploring the effects of TCM on microbiota in CIAD. Microbial diversity indices and abundance data were extracted. Meta-analysis was performed using R version 4.5.0 with the meta for package (version 5.0.1), and risk of bias was assessed using the Systematic Review Centre for Laboratory Animal Experimentation (SYRCLE) tool.

**Results:**

A total of 25 studies published from 2016 to 2025 were included, 20 of which were enrolled into the meta-analysis. No statistically significant overall effect of TCM on the examined gut or airway alpha-diversity indices was identified after re-analysis using REML random-effects models with Hartung–Knapp adjustment. Several pooled estimates showed substantial heterogeneity and sensitivity to individual-study omission. Most included studies reported differences in beta diversity between model and TCM groups. Changes in airway and gut microbial relative abundance were heterogeneous across studies. High or unclear risk of bias was identified in most SYRCLE domains for the included studies.

**Conclusion:**

Current preclinical evidence suggests that TCM may be associated with changes in airway and gut microbiota in CIAD animal models. However, the updated quantitative analyses did not demonstrate statistically significant overall effects on the alpha-diversity indices examined, and evidence concerning airway microbiota and microbial relative abundance remains heterogeneous. These findings should not be interpreted as evidence of microbiota restoration or therapeutic mechanisms. Well-designed studies with more standardized methodologies are warranted to clarify the impact of TCM on lung–gut axis microecology.

**Systematic Review Registration:**

https://www.crd.york.ac.uk/prospero/, identifier PROSPERO CRD420261385384.

## 1 Introduction

Chronic inflammatory airway disease (CIAD) encompasses a group of chronic respiratory disorders characterized by persistent airway inflammation, airflow limitation and airway remodeling (1). Chronic obstructive pulmonary disease (COPD) and bronchial asthma (BA) are the most common clinical phenotypes of CIAD. In 2019, the global prevalence of COPD among individuals aged 30–79 years was 10.3% (2). By 2060, more than 5.4 million people worldwide are projected to die from COPD and its related diseases (3). In 2021, the global number of asthma patients reached 262 million, resulting in 461,000 deaths, with the incidence showing an increasing trend each year (4). As major subtypes of CIAD, asthma and COPD have become a severe global public health burden and the leading causes of death among chronic respiratory illnesses (5). Accumulating evidence has demonstrated that microbiota play a critical role in the onset and progression of CIAD.

Studies have shown that alterations in the respiratory microbiota, such as the enrichment of *Haemophilus* and *Moraxella*, can activate neutrophil and eosinophil inflammation through toll-like receptor signaling pathways, thereby promoting an asthma phenotype in mouse models (6). The development of COPD is linked to an imbalance in Treg/Th17 cell immunity. Intestinal probiotics can attenuate the Th17 immune response while enhancing Treg cell numbers and function via elevated levels of short-chain fatty acids (SCFAs), thus suppressing the inflammatory response in COPD (7, 8). The concept of the “gut-lung axis” further expands the association between microbiota and respiratory diseases, confirming bidirectional regulatory interactions between the airway and gut microbiota. Through metabolic byproducts, immune modulation, and microbial migration, these interactions collectively contribute to the development of CIAD.

Traditional Chinese medicine (TCM) offers unique advantages in managing chronic respiratory diseases. Guided by its theoretical framework, therapeutic modalities such as oral herbal formulas, acupuncture, and tuina facilitate multi-level systemic regulation of the disease. TCM exerts therapeutic effects via anti-inflammatory, antioxidant and anti-airway remodeling mechanisms (9, 10), and exhibits prominent efficacy in regulating gut microbiota composition and their derived metabolites. Through the “lung-gut axis,” it enables immune regulation, thereby alleviating pulmonary inflammation. Thus, TCM provides novel insights and strategies for the management of CIAD.

Although substantial evidence has shown that TCM modulates microbiota in CIAD, existing studies are highly heterogeneous and lack systematic data synthesis. This systematic review and meta-analysis focuses on rodent models of CIAD, specifically addressing asthma and COPD phenotypes, with the goal of integrating the effects of TCM therapies on microbial diversity and abundance in oral cavity, airway and gut environments, thereby offering novel perspectives for CIAD treatment.

## 2 Methods

### 2.1 Protocol and registration

The protocol was registered on the PROSPERO platform (ID: CRD420261385384). The search report will adhered to the Preferred Reporting Items for Systematic reviews and Meta-Analyses (PRISMA) statement (11). A detailed PRISMA checklist can be found in **Supplementary Table 1**.

### 2.2 Information sources and search strategy

Two researchers (JS and HL) independently searched four databases: PubMed, EMBASE, the Cochrane Library, and Web of Science, covering the period from January 1, 2006, to January 5, 2026. Chinese-language databases were not searched. In addition, English-language limits were applied in the PubMed and Web of Science searches, as detailed in **Supplementary Table 2**. A combination of MeSH terms and free-text words was applied to ensure comprehensive coverage of relevant studies. The MeSH terms included ‘asthma’[MeSH Terms], ‘pulmonary disease, chronic obstructive’[MeSH Terms], ‘microbiota’[MeSH Terms], ‘Medicine, Chinese traditional’[MeSH Terms], ‘herbal medicine’[MeSH Terms], and ‘Medicine, Traditional’[MeSH Terms]. Appropriate Boolean operators (AND/OR) were used to construct search queries, and search strategies were tailored to each specific database. The wildcard term ‘*’ was used to improve search sensitivity. The complete search strategy is detailed in **Supplementary Table 2**.

### 2.3 Eligibility criteria

Studies were included if they fulfilled the following criteria: (1) Study type: animal experiments; (2) Study subjects: rodent models of asthma induced by stimuli such as ovalbumin, house dust mite, or ice water, or rodent models of COPD induced by cigarette smoke or other methods; (3) Interventions: TCM therapies, including Chinese herbal formulas (comprising at least two herbs, with no restrictions on dosage form), acupuncture, moxibustion, tuina etc.; (4) Comparators: at least one untreated blank model group or one positive control group treated with Western medicine; (5) Outcomes: studies conducting metagenomic or 16S rRNA sequencing analyses and providing microbial characteristics of the oral cavity, airway, or gut, including microbial diversity indices or relative abundance.

The following studies will be excluded: (1) Studies designed as *in vitro* or ex vivo experiments; (2) Use of non-rodent animals; (3) Interventions in the treatment group included single herbal medicine, herbal extracts, or Western medicines; (4) Comparators included TCM therapies; (5) Conference abstracts, reviews, letters, or articles for which full text was unavailable; (6) Studies from which effective data could not be extracted.

### 2.4 Study selection and data extraction

Two researchers (JS and HL) independently conducted literature screening and data extraction. Initial screening was carried out by examining titles and abstracts, and full-text evaluation was subsequently performed to select eligible studies according to predefined inclusion and exclusion criteria. All extracted data were cross-checked to guarantee accuracy and integrity. A standard data extraction form was applied to collect the following information: (1) study profiles, including title, first author, publication year, study design and sample type; (2) subject characteristics involving species, sample size; (3) animal model details, such as modeling method, dosage, administration route and exposure duration; (4) interventions regarding category, dosage, administration route and treatment course; (5) outcome measures. We mainly focused on microbial community composition in the oral cavity, airway and intestine of experimental animals, prioritizing alpha diversity, followed by beta diversity and microbial relative abundance at the phylum and genus levels; (6) sequencing parameters, including NGS platforms and amplified regions of 16S rRNA gene. Quantitative data of alpha diversity were retrieved from figures using Get Data Graph Digitizer. Discrepancies during the whole process were resolved via discussion with a third investigator (LC).

### 2.5 Risk of bias assessment

Two researchers (JS and HL) independently assessed the risk of bias using SYRCLE risk of bias tool (12). This tool, designed for assessing bias in intervention animal studies, comprises 10 items covering six types of bias: selection bias, performance bias, detection bias, attrition bias, reporting bias, and other sources of bias. Each SYRCLE item was judged as having a “low risk,” “high risk,” or “unclear risk” of bias. Items judged as “low risk” received a score of one; items judged as “unclear risk” or “high risk” received a score of zero. The total score for the 10 items was considered the quality score for each study. Any disagreements during the assessment process were resolved through consultation with a third researcher (LC).

### 2.6 Data synthesis and statistical analyses

Meta-analysis was performed using R version 4.5.0 with the meta for package (version 5.0.1). The mean difference (MD) and standard deviation (SD) of continuous variables included in the analysis were extracted. If the original studies provided medians and interquartile ranges, standardized formulas were applied to approximate the corresponding means and standard deviations, and the data were subsequently synthesized (13, 14). Differences in microbial alpha diversity between groups were quantified using the standardized mean difference (SMD) and 95% confidence intervals (CIs), and forest plots were generated. All alpha-diversity meta-analyses were conducted using random-effects models, with between-study variance estimated using restricted maximum likelihood (REML) and 95% confidence intervals calculated using the Hartung–Knapp adjustment. Heterogeneity was quantified using I² and τ². Disease-specific subgroup analyses were performed where data permitted. To explore heterogeneity in the primary intestinal model-versus-TCM comparisons, additional exploratory analyses were conducted by animal species and, where at least 10 studies were available, after restriction to studies using 16S rRNA sequencing of the V3–V4 region. Because all intestinal alpha-diversity comparisons were based on fecal samples, subgroup analysis by sample type was not informative. Leave-one-out sensitivity analyses were conducted for outcomes including at least five studies. For analyses involving at least 10 studies, publication bias was assessed using Egger’s test and funnel plots generated in Stata 17.0 (StataCorp LLC, College Station, TX, USA). P < 0.05 was considered indicative of potential publication bias, and the trim-and-fill method was applied where appropriate. To avoid double counting, when a study reported multiple eligible TCM intervention arms for the same outcome and comparison, these intervention arms were combined into a single TCM group before meta-analysis. The combined sample size was calculated as the sum of the sample sizes across the intervention arms, the combined mean was calculated as the sample-size-weighted mean, and the combined standard deviation was calculated by incorporating both within-group and between-group variability. A shared model or positive-control group was entered only once within each outcome-specific comparison and was not duplicated to match the number of intervention arms. Simpson-derived measures were eligible for quantitative pooling only when the reported metric and its direction could be verified to be comparable across studies; otherwise, they were not included in the quantitative synthesis.

## 3 Results

### 3.1 Study selection

The literature selection process is illustrated in **Figure 1**. A total of 292 records were identified through electronic database searches. After removing duplicates and screening titles and abstracts, 45 articles were selected for full-text review. Ultimately, 25 studies (15–39) were included in this meta-analysis. List of excluded studies following full-text review is provided in **Supplementary Table 3**.

**Figure 1:**
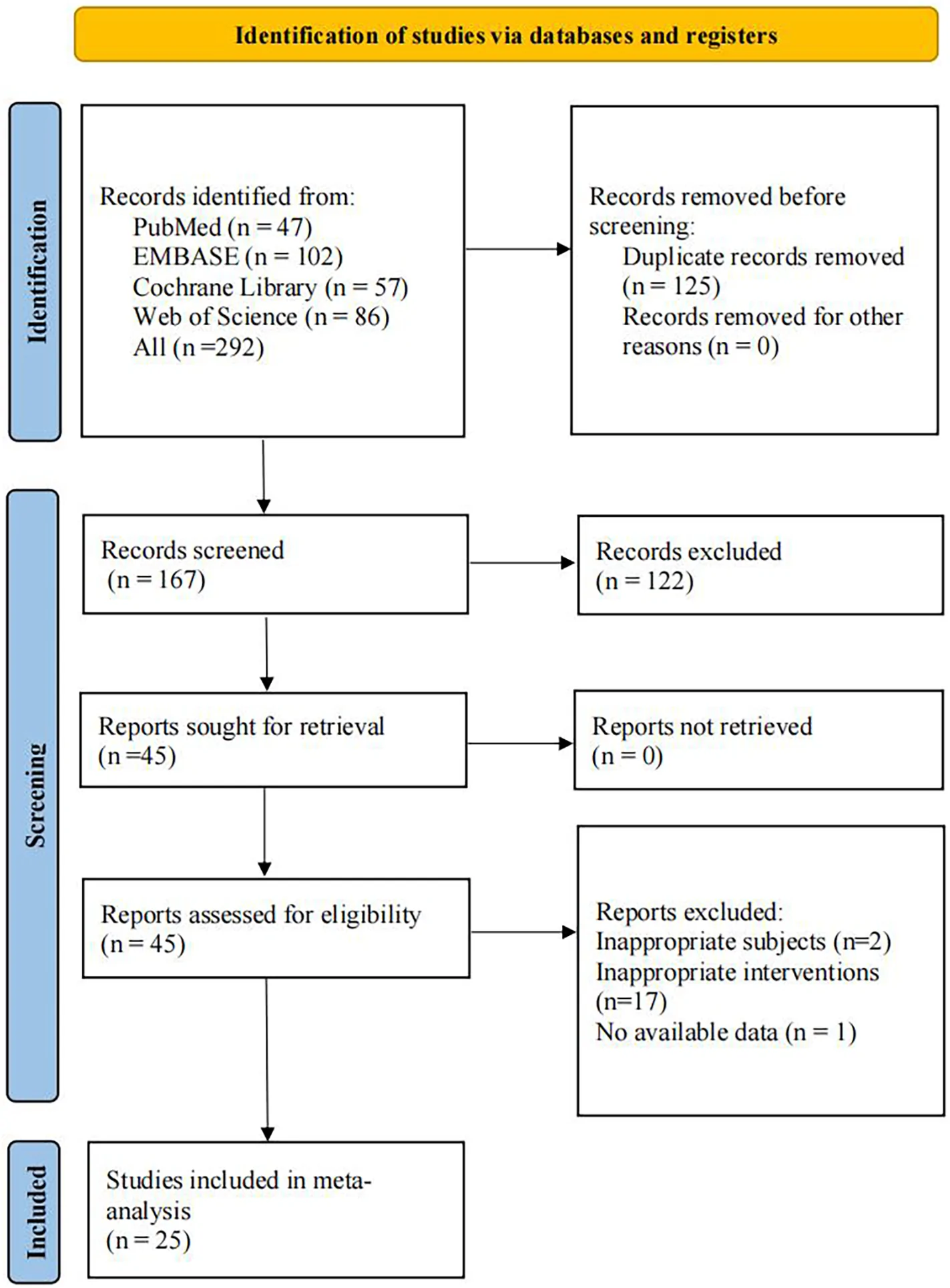
Flow chart diagram of study selection.

### 3.2 Study characteristics

Published between 2016 and 2025, all 25 studies were written in English. Of these, 10 focused on COPD and 15 on asthma. Among the studies, 14 used mice, 10 used rats, and one used guinea pigs as experimental subjects. COPD models were induced via cigarette smoke (n=1) or lipopolysaccharide combined with cigarette smoke (n=9). Asthma models were induced using ovalbumin (n=1), ovalbumin with aluminum hydroxide (n=9), ovalbumin plus aluminum hydroxide and pertussis toxoid (n=1), ovalbumin plus aluminum hydroxide and phosphate-buffered saline (n=2), ovalbumin plus aluminum hydroxide combined with ice-water swimming (n=1), or house dust mite (Der p) (n=1). Interventions included traditional Chinese medicine formulas (n=21), Chinese medicine drug pairs (n=2), and tuina therapy (n=2). The administration routes in the intervention group included intragastric gavage (n=19), oral administration (n=3), external therapy (n=2), and one was unspecified. Because 22 of the 25 studies used enteral administration (intragastric gavage or oral administration), whereas only two used external therapy and one did not report the route, the available evidence did not permit a meaningful comparison of microbiota outcomes by administration route. Twenty studies employed positive control interventions, such as dexamethasone (n=15), aminophylline (n=1), roflumilast (n=1), budesonide (n=1), methylprednisolone sodium succinate (n=1), and prednisone acetate combined with montelukast sodium (n=1). **Tables 1**, **2** summarize the characteristics of the included studies.

**Table 1:** General characteristics of the included studies.

Study (year)	Site	Type of sample	Design	Disease	NGS sequencing	Region	N_0_	N_1_	N_2_
Miaomiao Di (2025) (15)	Intestine	Fecal	RCT	COPD	Illumina MiSeq, 16S rRNA	V3-V4	6	6	6
Yanfei Hong (2025) (16)	Intestine	Fecal	RCT	BA	Illumina MiSeq, 16S rRNA	V3-V4	6	6	6
Yushan Zhao (2025) (17)	Intestine	Fecal	RCT	COPD	Illumina, 16S rRNA	V3-V4	6	6	6
Yanlin Li (2025) (18)	Intestine	Fecal	RCT	BA	16S rRNA	NR	6	6	6
Wenting Xu (2025) (19)	Intestine	Fecal	RCT	BA	Illumina, 16S rRNA	V3-V4	6	6	6
SiSi Han (2025) (20)	Intestine	Fecal	RCT	COPD	Illumina, 16S rRNA	V3-V4	3	3	3
Wei-Hsiang Hsu (2021) (21)	Intestine	Fecal	RCT	BA	Illumina, 16S rRNA	V3-V4	4	4	4
Yu Jia (2022) (22)	Intestine	Fecal	RCT	COPD	Illumina NovaSeq 6000, 16S rDNA	V3-V4	8	8	8
Yanhua Kong (2016) (23)	Intestine	Fecal	RCT	BA	16S rDNA	V3-V5	3	3	3
Yongan Wang (2021) (24)	Intestine	Fecal	RCT	COPD	16S rRNA	V3-V4	4-8	4-8	4-8
Yan Zhu (2020) (25)	Intestine	Fecal	RCT	BA	16S rRNA	V3-V4	5	5	5
Jing Mao (2022) (26)	Intestine	Fecal	RCT	COPD	16S rRNA	V3–V4	12	12	12
Fei Liu (2023) (27)	Intestine	Fecal	Unclear	BA	HiSeq, 16S rRNA	V3–V4	8	8	8
JunMei Wang (2023) (28)	Intestine	Fecal	RCT	COPD	Illumina PE250, 16S rDNA	NR	10	30	10
Rui He (2023) (29)	Intestine	Fecal	RCT	BA	Illumina, 16S rRNA	V3–V4	9	9	9
Yuan Ruan (2023) (30)	Intestine	Fecal	NR	BA	16S rDNA	V3–V4	8	8	8
Lin Lin (2025) (31)	Intestine	Fecal	RCT	BA	16S rDNA	V3–V4	5	5	5
Yuanyuan He (2022) (32)	Intestine	Fecal	RCT	BA	16S rRNA	V3–V4	15	15	15
Jiao Jiao (2022) (33)	Intestine	Fecal	RCT	COPD	Illumina, 16S rRNA	V3–V4	8	8	8
Haibo Hu (2023) (34)	Intestine	Fecal	RCT	BA	16S rRNA	V3–V4	6	6	6
Yuanyuan Li (2024) (35)	Intestine	Fecal	RCT	COPD	16S rRNA	V3–V4	5-6	5-6	5-6
Yantong Yu (2025) (36)	Intestine	Fecal	Unclear	BA	Illumina TruSeq, 16S rRNA	NR	6	12	6
Yanfei Hong (2025) (37)	Intestine	Fecal	RCT	BA	16S rRNA	V3–V4	24	24	24
Lingmiao Wen (2023) (38)	Airway	BALF	RCT	BA	MinION, 16S rDNA	full-length	8	24	8
Yueying Wu (2022) (39)	Airway	lung tissuel	RCT	COPD	Illumina NovaSeq, 16S rRNA	V3–V4	5	5	–

N_0_, Model group; N_1_, Intervention group; N_2_, Positive control group; “-”, None.

**Table 2:** Details on experimental designs of each included study.

Study(year)	Model induction				Intervention group			Positive control group		
	Rodent	Stimulatory factors	Route of administration	Duration	Intervention	Route of administration	Duration	Positive control	Route of administration	Duration
Miaomiao Di (2025) (15)	Mice	LPS+Cigarettes	Air exposure	8 weeks	Qi-Huo-Yi-Fei formula	i.g.	4 weeks	Dexamethasone	i.g.	4 weeks
Yanfei Hong (2025) (16)	Mice	OVA+Imject™ Aluminum	i.p.+Aerosol challenge	3 weeks	Ma-Xing-Shi-Gan decoction	i.g.	1 week	Dexamethasone	NR	1 week
Yushan Zhao (2025) (17)	Rats	LPS+Cigarettes	i.t.+Air exposure	16 weeks	Trichosanthis Pericarpium-Trichosanthis Radix herbal pair’s role	i.g.	4 weeks	Dexamethasone	i.g.	4 weeks
Yanlin Li (2025) (18)	Mice	OVA+Aluminum hydroxide	i.p.+Aerosol inhalation	4 weeks	Jian’er Qiangshen ointment	i.g.	3 weeks	Dexamethasone	i.g.	3 weeks
Wenting Xu (2025) (19)	Mice	OVA+Aluminum hydroxide	i.p.	6 weeks	Liujunzi decoction	i.g.	1 week	Dexamethasone	i.g.	1 week
SiSi Han (2025) (20)	Rats	LPS+Cigarettes	i.t.+Air exposure	12 weeks	Yi-Qi-Xuan-Fei formula	i.g.	4 weeks	–	–	–
Wei-Hsiang Hsu (2021) (21)	Mice	*Der p*-induced	i.t.	6 weeks	You-Gui-Wan	p.o.	26 days	Dexamethasone	p.o.	26 days
Yu Jia (2022) (22)	Rats	Cigarettes	Air exposure	4 weeks	Qibai Pingfei capsule	i.g.	2 weeks	–	–	–
Yanhua Kong (2016) (23)	Rats	OVA+Aluminum hydroxide+ Whooping cough toxoid	s.c.+Aerosol challenge	15 days	Recuperating lung decoction	i.g.	13 days	Dexamethasone	i.g.	13 days
Yongan Wang (2021) (24)	Mice	LPS+Cigarettes	i.n.	18 days	Xuanbai Chengqi decoction	i.g.	1 week	Dexamethasone	i.g.	1 week
Yan Zhu (2020) (25)	Rats	OVA+Al(OH)_3_	i.p.+Aerosol challenge	12 days	Chiropractic therapy	External therapy	3 weeks	–	–	–
Jing Mao (2022) (26)	Rats	LPS+Cigarettes	i.n.+Air exposure	8 weeks	Bufei Jianpi formula	p.o.	5 weeks	Aminophylline	p.o.	5 weeks
Fei Liu (2023) (27)	Mice	OVA+Aluminum hydroxide	i.p.+Aerosol challenge	2 weeks	Water extract of Pingchuan formula	i.g.	1 week	Dexamethasone	i.g.	1 week
JunMei Wang (2023) (28)	Rats	LPS+Cigarettes	i.n.+Air exposure	4 weeks	Seabuckthorn Wuwei pulvis	i.g.	2 weeks	Dexamethasone	i.g.	2 weeks
Rui He (2023) (29)	Rats	OVA+Aluminum hydroxide	i.p.+Aerosol challenge	8 weeks	Shaoyao-Gancao-Tang	i.g.	4 weeks	Dexamethasone	i.g.	4 weeks
Yuan Ruan (2023) (30)	Rats	OVA+Aluminum hydroxide	i.p.+Aerosol challenge	15 days	Tingli Dazao Xiefei decoction	i.g.	1 week	Dexamethasone	i.g.	1 week
Lin Lin (2025) (31)	Rats	OVA+Al(OH)_3_ gel	i.p.+Aerosol challenge	44 days	Traditional pediatric massage	External therapy	44 days	methylprednisolone sodium succinate	i.p. injection	22 days
Yuanyuan He (2022) (32)	Mice	OVA+Dry aluminum hydroxide+PBS	i.p.+Aerosol challenge	3 weeks	Xiaoning liquid	i.g.	1 week	Budesonide	Aerosol inhalation	1 week
Jiao Jiao (2022) (33)	Rats	LPS+Cigarettes	i.t.+Air exposure	4 weeks	Xuanbai Chengqi decoction	i.g.	2 weeks	–	–	–
Haibo Hu (2023) (34)	Mice	OVA and aluminum hydroxide (alum) mixture	i.p.+Aerosol challenge	6 weeks	Qing-Fei-Shen-Shi decoction	i.g.	4 weeks	Dexamethasone	i.g.	4 weeks
Yuanyuan Li (2024) (35)	Mice	LPS+Cigarettes	Instillation+Air exposure	60 days	Bufei Huoxue capsule	i.g.	30 days	Roflumilast	i.g.	30 days
Yantong Yu (2025) (36)	Rats	OVA+Aluminum hydroxide+Ice water swimming	i.p.+Aerosol challenge	23 days	Dry ginger and Schisandra chinensis	NR	NR	Dexamethasone	NR	NR
Yanfei Hong (2025) (37)	Mice	OVA+PBS+Imject™ aluminum	i.p.+Aerosol challenge	3 weeks	Tuo-Min-Ding-Chuan decoction	p.o.	2 weeks	Dexamethasone	p.o.	1 week
Lingmiao Wen (2023) (38)	Guinea pigs	OVA+Al(OH)3 suspension	i.m.+i.p.+Aerosol challenge	38 days	Modified Dingchuan decoction	i.g.	3 weeks	Prednisone acetate+Montelukast sodium	i.g.	1 time/day, 21 times
Yueying Wu (2022) (39)	Rats	LPS+cigarettes	Instillation+Air exposure	57 days	Yifei Sanjie formula	i.g.	27 dsys	–	–	–

NR, No Reported; i.p., Intraperitoneal injection; i.t., Intratracheal instillation; s.c., Subcutaneous injection; i.n., Intranasal instillation; i.m., Intramuscular injection; i.g., Intragastric gavage; p.o., Oral administration; “-”: None.

### 3.3 Risk of bias assessment

We assessed the risk of bias in the 25 studies using the SYRCLE checklist, with quality scores ranging from 2 to 6. Most studies showed a low risk of bias for items such as ‘random housing’ and ‘selective outcome reporting’. However, a high risk of bias was noted for ‘incomplete outcome data’. For items including ‘baseline characteristics’, ‘allocation concealment’, ‘blinding’, and ‘random outcome assessment’, the risk of bias was largely unclear. Baseline microbiota profiles and husbandry variables, including diet, water source, and housing conditions, were not reported consistently enough across studies for cross-study comparison. Future *in vivo* animal studies should place greater emphasis on random sequence generation, allocation concealment, and blinding of caregivers, investigators, and outcome assessors, as well as random outcome assessment. Developing more detailed and complete reporting standards is essential to enhance methodological reliability and the credibility of research findings. Detailed information is provided in **Supplementary Table 4**.

### 3.4 Sensitivity analysis and assessment of publication bias

Leave-one-out sensitivity analyses were conducted using the REML model with Hartung–Knapp adjustment. For intestinal model-versus-TCM comparisons, omission of Yanlin Li (18) changed the observed-species result from non-significant to significant (SMD 1.32, 95% CI 0.60 to 2.05), and omission of WenTing Xu (19) changed the Shannon result from non-significant to significant (SMD 1.09, 95% CI 0.08 to 2.11). In contrast, the non-significant Chao1 result was not changed by omission of any single study. For the TCM-versus-positive-control comparison, omission of Yantong Yu (36) changed the Shannon result to statistical significance (SMD 0.56, 95% CI 0.06 to 1.05). No single-study omission changed the overall inference for airway Chao1. These findings indicate that several pooled estimates were sensitive to individual-study inclusion and should be interpreted cautiously. Details are provided in **Supplementary Figures 1**–**3**. Egger’s test showed no statistically significant evidence of publication bias for outcomes including at least 10 studies (both P > 0.05; **Supplementary Figure 4**).

### 3.5 Effects of TCM on the microbiota

#### 3.5.1 Effects of TCM on microbial alpha diversity

This study compared differences in microbial alpha diversity between the model group and the TCM intervention group. Quantitative synthesis was performed for observed species, Chao1, and Shannon indices. A total of 20 eligible studies were ultimately enrolled in the present meta-analysis. Among them, 6 studies (18, 25, 28, 31, 33, 36) reported observed species, 13 studies (17–19, 22, 25–28, 30, 32, 33, 36, 37) reported the Chao1 index, 13 studies (17–19, 21, 25–28, 32–34, 36, 37) reported the Shannon index. Seven studies reported Simpson-derived measures; however, these data were not included in the quantitative synthesis because their definitions and directions were not consistently verifiable across studies. Six studies provided data on airway microbiota alpha diversity: 5 studies (19, 22, 33, 34, 39) reported the Chao1 index, and 4 studies (19, 33, 38, 39) reported the Shannon index.

For gut microbiota profiling, REML random-effects meta-analyses with Hartung–Knapp adjustment showed no statistically significant differences between the TCM and model groups for observed species (SMD 0.91, 95% CI -0.49 to 2.31; I²=79.8%), Chao1 (SMD 0.55, 95% CI -0.68 to 1.78; I²=91.7%), or Shannon (SMD 0.78, 95% CI -0.43 to 1.99; I² = 92.0%). Disease-specific estimates were also non-significant after the revised analysis. In exploratory analyses by animal species, the between-species difference was statistically significant for Chao1 (P = 0.032), but substantial heterogeneity remained within both the mouse and rat subgroups. No statistically significant between-species difference was identified for Shannon (P = 0.184). The exploratory species analyses were limited to mouse and rat studies because only one included study used guinea pigs and the available outcome-specific data were insufficient for a separate guinea pig analysis. Restriction to studies using 16S rRNA sequencing of the V3-V4 region did not materially alter the non-significant results for Chao1 (SMD 0.63, 95% CI -1.03 to 2.29) or Shannon (SMD 0.76, 95% CI -0.69 to 2.20). Detailed information is presented in **Figure 2**.

**Figure 2:**
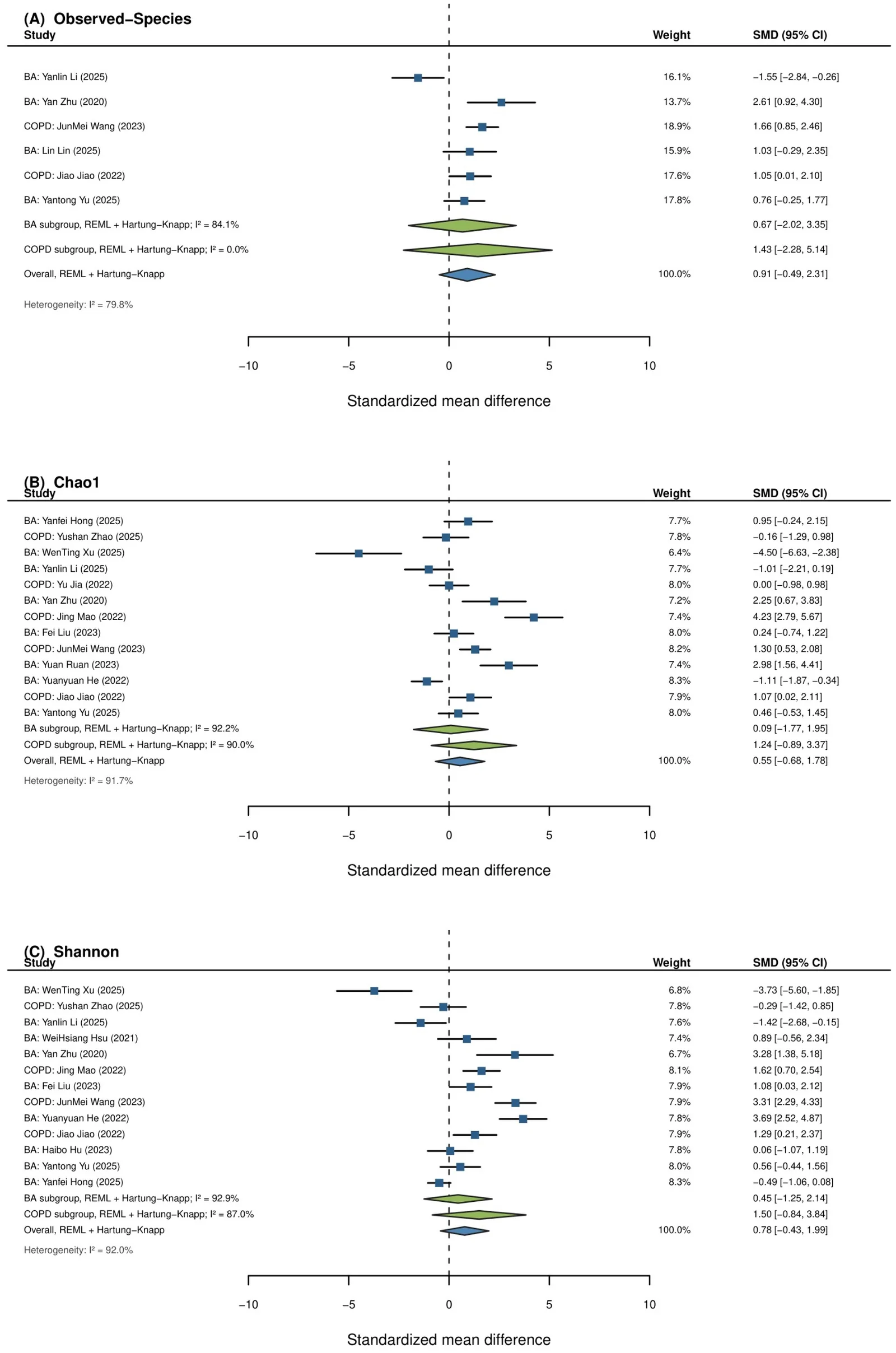
Forest plots of alpha-diversity indices of intestinal microbiota among the model and TCM intervention groups. **(A)** Observed-Species; **(B)** Chao1; **(C)** Shannon.

For airway microbiota, REML random-effects meta-analyses with Hartung–Knapp adjustment showed no statistically significant differences between the TCM and model groups for the Chao1 index (SMD −0.91, 95% CI −2.71 to 0.90; I² = 83.4%) or Shannon index (SMD −0.35, 95% CI −5.03 to 4.34; I² = 94.9%). Because only four to five studies were available for these outcomes, additional subgroup analyses by animal species, sample type, or sequencing approach were not sufficiently informative to explain the heterogeneity. These sparse data also did not allow a formal comparison of the effects of enteral administration on airway versus gut microbiota. Detailed information is presented in **Figure 3**.

**Figure 3:**
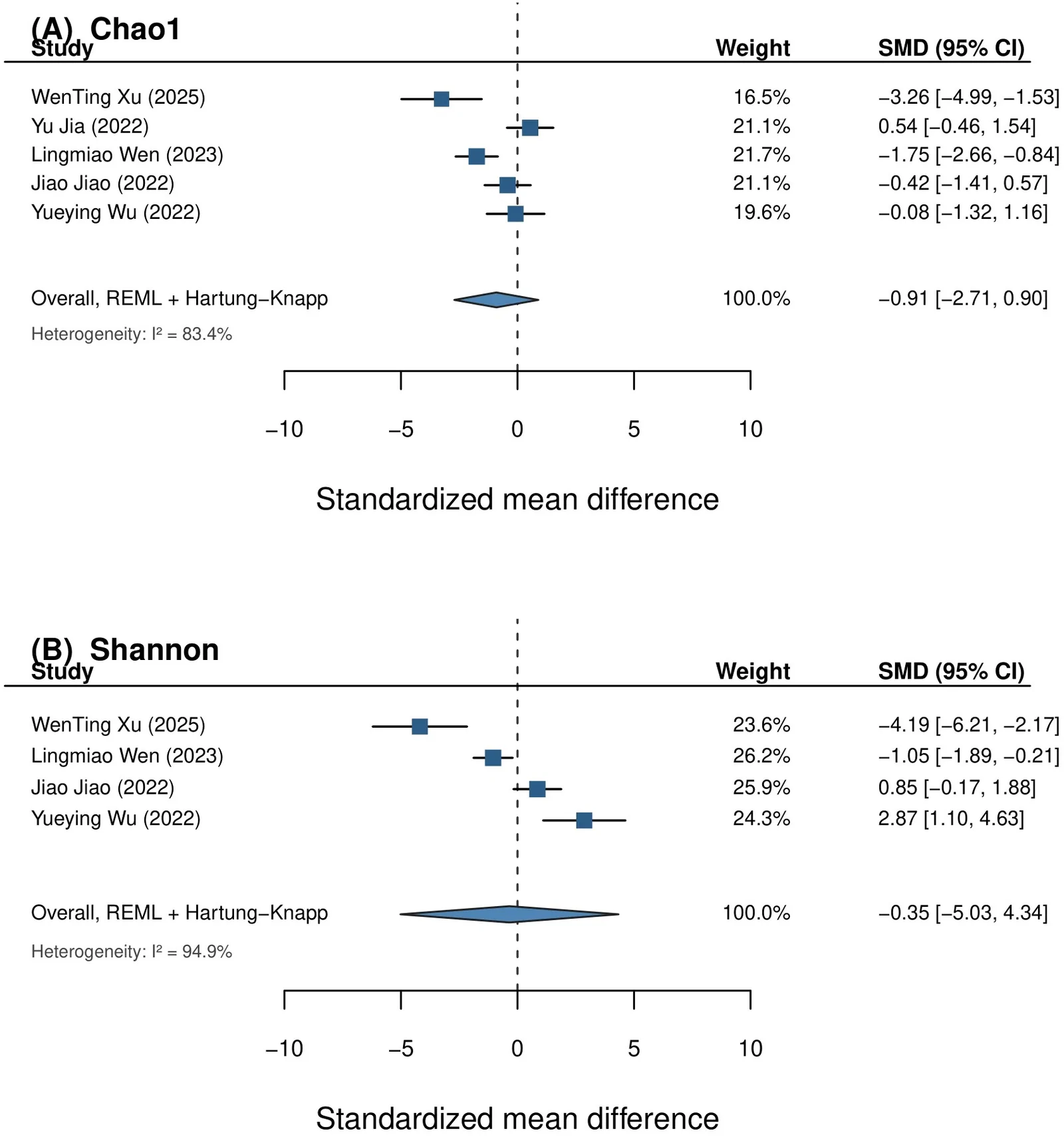
Forest plots of alpha diversity indices of airway microbiota among model and TCM intervention groups. **(A)** Chao1; **(B)** Shannon.

Additionally, we included Western medicine as a positive control group to compare its efficacy with TCM. Nine studies provided gut microbiota data: 3 studies (28, 31, 36) reported species richness, 7 studies (17, 19, 26–28, 32, 35) reported the Chao1 index, and 6 studies (16, 19, 27, 28, 32, 36) reported the Shannon index. In the REML random-effects analyses with Hartung–Knapp adjustment, no statistically significant differences were observed between the TCM and positive-control groups for intestinal observed species (SMD 0.21, 95% CI −0.76 to 1.18), Chao1 (SMD 0.42, 95% CI −0.55 to 1.38; I² = 78.2%), or Shannon (SMD 0.42, 95% CI −0.15 to 0.99). For airway microbiota, the corresponding analyses also showed no statistically significant differences for Chao1 (SMD 0.51, 95% CI −4.62 to 5.63) or Shannon (SMD −0.05, 95% CI −6.54 to 6.44). Detailed information is presented in **Supplementary Figures 5**, **6**.

#### 3.5.2 Effects of TCM on microbial beta diversity

Among all included studies, 15 reported beta diversity results, with the most commonly used measurement methods being PCoA based on weighted and unweighted UniFrac distances, Jaccard distances, and Bray-Curtis dissimilarity. In gut microbiota studies, four studies (15, 22, 24, 28) on COPD found compositional differences between the TCM group and the COPD model group, whereas one study (35) detected no significant difference, and another (33) showed that the microbial structure of the TCM group tended toward that of the healthy group. For asthma, four studies (23, 25, 30, 37) reported compositional differences between the TCM group and the asthma model group, one study (16) found no significant difference, and seven studies (19, 23, 25, 29, 30, 32, 34) indicated that the TCM group approached the normal group in microbial structure. In airway microbiota studies, one study (22) on COPD reported compositional differences between the TCM group and the COPD model group, and another (33) showed that the microbial structure of the TCM group tended toward that of the healthy group. For asthma, one study (19) suggested that the TCM group reduced the compositional difference compared with the healthy group. Detailed information is presented in **Supplementary Table 5**.

#### 3.5.3 Effects of TCM on microbial taxa abundance

Regarding microbial community abundance, most included studies indicated that the airway and gut microbiota structures in animal models of chronic airway diseases (asthma and COPD) were significantly disrupted. TCM interventions were associated with changes in microbial community structure and relative abundance compared with the model groups. Studies on COPD primarily focused on changes in the phylum-level composition of airway and gut microbiota. Results showed that the dominant gut microbiota in normal rats included *Bacteroidetes*, *Firmicutes*, *Proteobacteria*, and *Fusobacteria* (22, 26). Following induction with lipopolysaccharide and cigarette smoke, the relative abundances of *Firmicutes*, *Fusobacteria*, and *Actinobacteria* decreased in the COPD model group, while those of *Bacteroidetes*, *Tenericutes*, *Verrucomicrobia*, and *Desulfobacterota* increased (22, 26, 28, 35). However, heterogeneity was observed among the few included studies; one study found a decreasing trend in the abundance of *Proteobacteria* in the model group (28), while another study reported an increased proportion of *Proteobacteria* (26). Following TCM intervention, changes in the relative abundance of these taxa were reported; however, the directions of these changes were not fully consistent across studies and cannot establish restoration of microbial homeostasis. In terms of airway microbiota, the top five dominant phyla were *Bacteroidetes*, *Proteobacteria*, *Firmicutes*, *Tenericutes*, and *Actinobacteria*. After modeling, the proportions of *Bacteroidetes*, *Proteobacteria*, *Firmicutes*, and *Actinobacteria* decreased, while the proportion of *Tenericutes* increased (22). Compared with the model group, one study reported changes in the relative abundance of *Pseudomonas*, *Microbacterium*, and *Allobaculum* at the genus level (33).

Research on asthma primarily focused on the structural changes of the gut microbiota, with changes at the phylum level centered on *Firmicutes*, *Bacteroidetes*, and their ratio (F/B ratio). Compared with the healthy group, the relative abundance of *Bacteroidetes* was lower and that of *Firmicutes* was higher in the model group (25, 37), resulting in a significantly elevated F/B ratio. In the reported studies, the TCM group showed a lower F/B ratio than the model group, while no statistically significant difference was observed between the TCM and blank groups in two reports (21, 34). However, some study conclusions were heterogeneous. One study showed that ovalbumin (OVA) exposure did not cause significant changes in the relative abundance of major bacterial phyla but decreased the abundance of the class *Fusobacteriia* and increased the abundances of the class *Bacilli*, family *Lactobacillaceae*, and genus *Lactobacillus* (31). In contrast, one study reported increased relative abundances of *Lactococcus*, *Lactobacillus*, and *Limosilactobacillus* following TCM intervention (16). Another study reported that in the OVA-induced asthma model, the abundance of Bacteroidetes was significantly higher than that of *Firmicutes* compared with the TCM group (18). Detailed information is provided in **Supplementary Table 6**.

## 4 Discussion

Although previous studies have extensively explored the regulatory effects of TCM on the microbiota in chronic inflammatory airway diseases, this study is the first to systematically integrate evidence on the diversity and abundance of airway and gut microbiota in asthma and COPD rodent models. Based on published evidence, we hypothesized that TCM would increase alpha diversity of the airway and gut microbiota in CIAD. However, after re-analysis using REML random-effects models with Hartung–Knapp adjustment, no statistically significant overall differences were observed between the TCM and model groups for the intestinal observed-species, Chao1, or Shannon indices, or for the airway Chao1 and Shannon indices. The direction and magnitude of the estimates varied across studies, and substantial heterogeneity was present for most outcomes. Simpson-derived measures were not quantitatively pooled because their reporting definitions and directions could not be verified consistently across studies. Given the current evidence base, particularly the scarcity of airway microbiota studies, the findings should be interpreted cautiously.

Changes in microbial alpha diversity are commonly used to assess dysbiosis, primarily quantified through richness and evenness (40). Among these indices, observed species and Chao1 focus on species richness, while the Shannon and Simpson indices capture both richness and evenness (41). However, higher diversity does not necessarily indicate a ‘better’ or ‘healthier’ state, and its implications must be interpreted within the context of the community environment and underlying mechanisms. The included studies showed considerable heterogeneity. In leave-one-out analyses, omission of Yanlin Li (18) changed the intestinal observed-species result from non-significant to significant, and omission of WenTing Xu (19) changed the intestinal Shannon result from non-significant to significant; the intestinal Chao1 result was not changed by omission of any single study. For the TCM-versus-positive-control comparison, omission of Yantong Yu (36) changed the Shannon result to statistical significance. These findings indicate that several pooled estimates were sensitive to individual-study inclusion. Exploratory analyses identified a statistically significant between-species difference for Chao1, although substantial heterogeneity remained within both mouse and rat subgroups. Restriction to studies using the V3–V4 region did not materially alter the non-significant Chao1 or Shannon results. Because all intestinal alpha-diversity comparisons used fecal samples and only four to five airway studies were available, further subgroup analyses by sample type or sequencing approach were not informative. Moreover, the magnitude and direction of reported gut microbiota changes differed between COPD and asthma studies. Although COPD and asthma have different pathophysiology and reported patterns of gut dysbiosis (42–44), the limited number of studies and substantial heterogeneity preclude firm conclusions regarding disease-specific effects of TCM on the microbiota. We further compared the efficacy of TCM and Western medicine interventions on airway and gut alpha diversity and observed no statistically significant differences between the two groups.

Beta diversity serves as a core indicator characterizing compositional structure and species turnover and differentiation across samples and groups, quantifying similarity or dissimilarity of community composition between groups (45). In this meta-analysis, the 15 included studies predominantly used principal component analysis (PCA), principal coordinate analysis (PCoA), and non-metric multidimensional scaling (NMDS) for visualizing community structure, along with statistical tests such as permutational multivariate analysis of variance (PERMANOVA) and analysis of similarities (ANOSIM). Most studies observed significant separation of microbial community structures between disease model and normal control groups, confirming that disease states can alter the overall community structure of airway or gut microbiota. Several studies reported that community structures in TCM-treated animals were closer to those of healthy controls than to those of model animals. However, because the included studies used different sampling sites, distance metrics, sequencing approaches, and statistical tests, beta-diversity findings were not quantitatively pooled and no consistent pattern could be established. Additional clinical and experimental heterogeneity, including differences in animal species, disease-induction methods, intervention composition and duration, and timing of sample collection, may also have contributed to the inconsistent beta-diversity findings. Accordingly, these observations provide complementary information on compositional differences but cannot, by themselves, demonstrate restoration of microbial homeostasis.

Furthermore, based on data from the included studies, we specifically compared community abundance between the model group and the TCM intervention group. We found that changes in relative community abundance were not entirely consistent. These observations were based on qualitative reporting across heterogeneous studies and should not be interpreted as demonstrating restoration of microbial homeostasis or a therapeutic mechanism of TCM. The alpha-diversity indices in this review reflect the richness and evenness of the overall microbial community, rather than the diversity or richness of pre-defined beneficial or potentially pathogenic taxa. Although several studies reported changes in the relative abundance of taxa that have been associated with pathogenicity in particular contexts, these data were not sufficiently consistent to support a separate quantitative synthesis of potentially pathogenic taxa. *Firmicutes*, *Bacteroidetes*, *Actinobacteria*, and *Proteobacteria* constituted the core communities in healthy lungs and intestines. For COPD, we synthesized the findings from the included studies and observed that TCM could increase the abundance of these phyla in the airways while decreasing that of Tenericutes. Moreover, TCM could increase the abundance of *Actinobacteria*, *Bacteroidetes*, and *Fusobacteria* in the intestines. Although the reported changes in *Firmicutes* varied across studies, no consistent direction of effect could be established. At the genus level, changes in the abundance of several genera were consistent. Individual studies reported increases in the relative abundance of *Bifidobacterium, Lactobacillus, Faecalibacterium, Blautia, and Butyricimonas*, and decreases in *Ruminococcus, Roseburia, Ralstonia, and Mycoplasma*. Regarding the gut microbiota in asthma, the effects on *Firmicutes* and *Bacteroidetes* were heterogeneous. The reported findings included a lower *Firmicutes/Bacteroidetes* ratio and differences in the relative abundance of *Lactobacillus, Lactococcus, Ruminococcus torques, Pseudomonas, and Streptococcus*. *Firmicutes* and *Bacteroidetes* dominate dietary fiber fermentation, promoting regulatory T cell differentiation through SCFAs production, thereby influencing immune responses and inflammatory reactions (46). Among these, propionate and butyrate are critical for maintaining intestinal barrier integrity and immune homeostasis and can coordinately block NF-κB p65 subunit translocation, significantly downregulating pro-inflammatory factors such as TNF-α and IL-6 (47, 48). However, in our study, changes in *Firmicutes* showed significant heterogeneity, which may relate to its bidirectional regulatory role. Besides producing SCFAs, some opportunistic pathogenic bacteria possess pro-inflammatory effects, harboring structural elements such as polysaccharide capsules, fimbriae, and M proteins that can promote airway inflammation and various respiratory diseases (49). *Lactobacillus* and *Bacteroides* are common beneficial SCFA-producing genera and are significantly negatively correlated with inflammatory factors, whereas opportunistic pathogens like *Ralstonia* and *Mycoplasma* correlate positively with these factors (39). *Proteobacteria* include several potential pathogens. These bacteria produce lipopolysaccharides, proteases, and toxins that directly stimulate and damage the airway epithelium (50), promoting CIAD development.

These qualitative observations should not be interpreted as demonstrating that TCM exerts therapeutic effects through a defined lung–gut-axis mechanism. The present review did not pool functional metagenomic, metabolomic, host-barrier, or signaling-pathway outcomes; therefore, changes in taxonomic relative abundance alone cannot establish microbial function, causality, or restoration of microbial homeostasis. Previous experimental studies have proposed associations between gut microbiota, microbial metabolites, inflammation, epithelial barrier function, and signaling pathways (51–59), but these pathways require direct and standardized validation in future CIAD animal studies.

This meta-analysis synthesized available evidence on airway and gut microbiota in asthma and COPD animal models. The updated quantitative analyses did not identify a statistically significant overall effect of TCM on the alpha-diversity indices examined. Qualitative findings on microbial relative abundance were heterogeneous and do not establish restoration of microbial homeostasis or therapeutic mechanisms. However, several limitations of this study should be noted. First, the sample sizes of the included animal studies were relatively small, and there were few studies investigating airway microbiota in asthma and COPD, which limited the statistical power. Second, all data were derived from 16S rRNA sequencing, which constrained interpretations at the species level. Third, the included studies showed methodological limitations and substantial heterogeneity, and several pooled estimates were sensitive to omission of individual studies. Fourth, potential confounding variables, including modeling methods, sampling sites and types, sequencing platforms, and administration duration, may have introduced bias and affected the interpretation of results. Fifth, the use of medians and interquartile ranges to estimate means and standard deviations based on figures provided in the literature may have introduced minor errors in the analysis of alpha diversity indices. Sixth, Chinese-language databases were not searched, and English-language limits were applied in part of the search strategy. Relevant studies published in Chinese or indexed exclusively in Chinese databases may therefore have been missed, potentially introducing language and database bias and limiting the completeness of the available evidence. Seventh, most interventions were administered by intragastric gavage or orally, and the limited number of non-enteral studies precluded route-specific comparisons. In addition, airway microbiota alpha-diversity data were available for only four to five studies per outcome; therefore, this review could not determine whether enteral TCM administration produces differential effects in gut and airway microbiota or demonstrate a causal gut-lung axis. Eighth, baseline microbiota profiles and husbandry variables, including diet, water source, and housing conditions, were not reported consistently across the included studies. These factors may influence microbial composition and could not be evaluated as effect modifiers in the present review.

## 5 Conclusion

In summary, the current evidence suggests that TCM may influence the diversity and abundance of airway and gut microbiota in animal models of CIAD; however, the updated quantitative analyses did not demonstrate statistically significant overall effects on the alpha-diversity indices examined. The evidence base is limited by substantial heterogeneity, small sample sizes, and sensitivity of some pooled estimates to individual-study inclusion. Future well-designed studies using standardized sampling, sequencing, and analytical methods are needed to clarify the effects of TCM on the CIAD microbiota.

## Data Availability

The original contributions presented in the study are included in the article/**Supplementary Material**. Further inquiries can be directed to the corresponding author.
