# Supplementary material for: Modulation of microbiota by traditional Chinese medicine in chronic inflammatory airway disease: a systematic review and meta-analysis of animal studies
Source: Front Immunol. 2026 Sep 30;17:1904457. doi: 10.3389/fimmu.2026.1904457 (PMC13661446; doi:10.3389/fimmu.2026.1904457)
Supplement: Supplementary file 1 [file SupplementaryFile1.docx]

Supplementary Material

# 1 Supplementary Figures and Tables

## 1.1 Supplementary Figures

**Supplementary Figure S1.** Sensitivity analysis of the gut microbiomes between model group and the intervention groups. (A) Chao1; (B) Observed-Species; (C) Shannon. The shaded band indicates the 95% CI of the pooled estimate from the full analysis, the solid blue line indicates the corresponding pooled SMD, and the dashed red line indicates the line of no effect.

**Supplementary Figure S2.** Sensitivity analysis of gut microbiomes between intervention and positive control groups. (A) Chao1; (B) Shannon. The shaded band indicates the 95% CI of the pooled estimate from the full analysis, the solid blue line indicates the corresponding pooled SMD, and the dashed red line indicates the line of no effect.

**Supplementary Figure S3.** Sensitivity analysis of the Chao1 index for airway microbiomes between model and intervention groups. The shaded band indicates the 95% CI of the pooled estimate from the full analysis, the solid blue line indicates the corresponding pooled SMD, and the dashed red line indicates the line of no effect.

**Supplementary Figure S4.** Egger’s test and funnel plot for publication bias. (A) Chao1 Egger’s test; (B) Shannon Egger’s test; (C) Chao1 funnel plot; (D) Shannon funnel plot.

**Supplementary Figure S5.** Comparison of gut microbial alpha diversity between intervention and positive control groups. (A) Observed-Species; (B) Chao1; (C) Shannon.

**Supplementary Figure S6.** Comparison of airway microbial alpha diversity between intervention and positive control groups. (A) Chao1; (B) Shannon.

## 1.2 Supplementary Tables

**Supplementary Table S1.** PRISMA 2020 Checklist.

| **Section and Topic** | **Item #** | **Checklist item** | **Location where item is reported** |
| --- | --- | --- | --- |
| **TITLE** | | |  |
| Title | 1 | Identify the report as a systematic review. | Manuscript, page 1. |
| **ABSTRACT** | | |  |
| Abstract | 2 | See the PRISMA 2020 for Abstracts checklist. | Manuscript, pages1-2. |
| **INTRODUCTION** | | |  |
| Rationale | 3 | Describe the rationale for the review in the context of existing knowledge. | Manuscript, page 2. |
| Objectives | 4 | Provide an explicit statement of the objective(s) or question(s) the review addresses. | Manuscript, page 2. |
| **METHODS** | | |  |
| Eligibility criteria | 5 | Specify the inclusion and exclusion criteria for the review and how studies were grouped for the syntheses. | Manuscript, page 3. |
| Information sources | 6 | Specify all databases, registers, websites, organisations, reference lists and other sources searched or consulted to identify studies. Specify the date when each source was last searched or consulted. | Manuscript, page 3. |
| Search strategy | 7 | Present the full search strategies for all databases, registers and websites, including any filters and limits used. | Supplementary Material, pages 11-18, Table S2. |
| Selection process | 8 | Specify the methods used to decide whether a study met the inclusion criteria of the review, including how many reviewers screened each record and each report retrieved, whether they worked independently, and if applicable, details of automation tools used in the process. | Manuscript, pages 3-4. |
| Data collection process | 9 | Specify the methods used to collect data from reports, including how many reviewers collected data from each report, whether they worked independently, any processes for obtaining or confirming data from study investigators, and if applicable, details of automation tools used in the process. | Manuscript, pages 3-4. |
| Data items | 10a | List and define all outcomes for which data were sought. Specify whether all results that were compatible with each outcome domain in each study were sought (e.g. for all measures, time points, analyses), and if not, the methods used to decide which results to collect. | Manuscript, pages 3-4. |
|  | 10b | List and define all other variables for which data were sought (e.g. participant and intervention characteristics, funding sources). Describe any assumptions made about any missing or unclear information. | Manuscript, pages 3-4, 17-22 and Tables 1-2. |
| Study risk of bias assessment | 11 | Specify the methods used to assess risk of bias in the included studies, including details of the tool(s) used, how many reviewers assessed each study and whether they worked independently, and if applicable, details of automation tools used in the process. | Manuscript, page 4; Supplementary Material, pages 21-23, Table S4. |
| Effect measures | 12 | Specify for each outcome the effect measure(s) (e.g. risk ratio, mean difference) used in the synthesis or presentation of results. | Manuscript, page 4. |
| Synthesis methods | 13a | Describe the processes used to decide which studies were eligible for each synthesis (e.g. tabulating the study intervention characteristics and comparing against the planned groups for each synthesis (item #5)). | Manuscript, page 4. |
|  | 13b | Describe any methods required to prepare the data for presentation or synthesis, such as handling of missing summary statistics, or data conversions. | Manuscript, page 4. |
|  | 13c | Describe any methods used to tabulate or visually display results of individual studies and syntheses. | Manuscript, page 4. |
|  | 13d | Describe any methods used to synthesize results and provide a rationale for the choice(s). If meta-analysis was performed, describe the model(s), method(s) to identify the presence and extent of statistical heterogeneity, and software package(s) used. | Manuscript, page 4. |
|  | 13e | Describe any methods used to explore possible causes of heterogeneity among study results (e.g. subgroup analysis, meta-regression). | Manuscript, page 4 |
|  | 13f | Describe any sensitivity analyses conducted to assess robustness of the synthesized results. | Manuscript, pages 5-6. Supplementary Material, pages 1-3, Figures S1-S3. |
| Reporting bias assessment | 14 | Describe any methods used to assess risk of bias due to missing results in a synthesis (arising from reporting biases). | Manuscript, page 4. Supplementary Material, page 3, Figure S4. |
| Certainty assessment | 15 | Describe any methods used to assess certainty (or confidence) in the body of evidence for an outcome. | Not reported. |
| **RESULTS** | | |  |
| Study selection | 16a | Describe the results of the search and selection process, from the number of records identified in the search to the number of studies included in the review, ideally using a flow diagram. | Manuscript, page 5 and Figure 1; Supplementary Material, pages 12-19, Table S2. |
|  | 16b | Cite studies that might appear to meet the inclusion criteria, but which were excluded, and explain why they were excluded. | Manuscript, page 5; Supplementary Material, pages 20-21, Table S3. |
| Study characteristics | 17 | Cite each included study and present its characteristics. | Manuscript, pages 5, 17-22 and Tables 1-2. |
| Risk of bias in studies | 18 | Present assessments of risk of bias for each included study. | Manuscript, page 5, Supplementary Material,  pages 22-24, Table S4. |
| Results of individual studies | 19 | For all outcomes, present, for each study: (a) summary statistics for each group (where appropriate) and (b) an effect estimate and its precision (e.g. confidence/credible interval), ideally using structured tables or plots. | Manuscript, pages 6-8, 22 and Figures 2-3; Supplementary Material, pages 4-5, Figures S5-S6. |
| Results of syntheses | 20a | For each synthesis, briefly summarise the characteristics and risk of bias among contributing studies. | Manuscript, pages 5-6,  Supplementary Material, page 3, Figures S4. |
|  | 20b | Present results of all statistical syntheses conducted. If meta-analysis was done, present for each the summary estimate and its precision (e.g. confidence/credible interval) and measures of statistical heterogeneity. If comparing groups, describe the direction of the effect. | Manuscript, pages 6-8, 22 and Figures 2-3; Supplementary Material, pages 4-5, Figures S5-S6. |
|  | 20c | Present results of all investigations of possible causes of heterogeneity among study results. | Manuscript, pages 6-9 |
|  | 20d | Present results of all sensitivity analyses conducted to assess the robustness of the synthesized results. | Manuscript, pages 5-6; Supplementary Material, pages 1-2, Figures S1-S3. |
| Reporting biases | 21 | Present assessments of risk of bias due to missing results (arising from reporting biases) for each synthesis assessed. | Manuscript, page 5; Supplementary Material, page 3, Figures S4. |
| Certainty of evidence | 22 | Present assessments of certainty (or confidence) in the body of evidence for each outcome assessed. | Not reported. |
| **DISCUSSION** | | |  |
| Discussion | 23a | Provide a general interpretation of the results in the context of other evidence. | Manuscript, pages 8-10. |
|  | 23b | Discuss any limitations of the evidence included in the review. | Manuscript, page 10. |
|  | 23c | Discuss any limitations of the review processes used. | Manuscript, page 10. |
|  | 23d | Discuss implications of the results for practice, policy, and future research. | Manuscript, page 10. |
| **OTHER INFORMATION** | | |  |
| Registration and protocol | 24a | Provide registration information for the review, including register name and registration number, or state that the review was not registered. | Manuscript, page 3; PROSPERO CRD420261385384. |
|  | 24b | Indicate where the review protocol can be accessed, or state that a protocol was not prepared. | Manuscript, pages 2; PROSPERO CRD420261385384. |
|  | 24c | Describe and explain any amendments to information provided at registration or in the protocol. | Manuscript, pages 2; PROSPERO CRD420261385384. |
| Support | 25 | Describe sources of financial or non-financial support for the review, and the role of the funders or sponsors in the review. | Manuscript, page 11. |
| Competing interests | 26 | Declare any competing interests of review authors. | Manuscript, page 11. |
| Availability of data, code and other materials | 27 | Report which of the following are publicly available and where they can be found: template data collection forms; data extracted from included studies; data used for all analyses; analytic code; any other materials used in the review. | Manuscript, page 11; Supplementary Material, pages 1-43. |

**Supplementary Table S2.** The details of the search strategy.

1.1 **Pubmed search stratergy for 47**

| **Search** | **Hits** |
| --- | --- |
| #1  "asthma"[MeSH Terms] OR "asthma"[Title/Abstract] OR "asthmas"[Title/Abstract] OR "asthma, bronchial"[Title/Abstract] OR "bronchial asthma"[Title/Abstract] OR "pulmonary disease, chronic obstructive"[MeSH Terms] OR "chronic obstructive pulmonary diseases"[Title/Abstract] OR "COPD"[Title/Abstract] OR "chronic obstructive lung disease"[Title/Abstract] OR "chronic obstructive pulmonary disease"[Title/Abstract] OR "COAD"[Title/Abstract] OR "chronic obstructive airway disease"[Title/Abstract] OR "airflow obstruction, chronic"[Title/Abstract] OR "airflow obstructions, chronic"[Title/Abstract] OR "chronic airflow obstructions"[Title/Abstract] OR "chronic airflow obstruction"[Title/Abstract] OR "chronic inflammatory airway disease"[Title/Abstract] OR "chronic inflammatory airway diseases"[Title/Abstract] | 323847 |
| #2  "Microbiota"[MeSH Terms] OR "Microbiota"[Title/Abstract] OR "Microbial Community"[Title/Abstract] OR "Microbiotas"[Title/Abstract] OR "community microbial"[Title/Abstract] OR "Microbial Communities"[Title/Abstract] OR "Microbial Community Composition"[Title/Abstract] OR "community composition microbial"[Title/Abstract] OR "composition microbial community"[Title/Abstract] OR "Microbial Community Compositions"[Title/Abstract] OR "Microbiome"[Title/Abstract] OR "Microbiomes"[Title/Abstract] OR "Microbial Community Structure"[Title/Abstract] OR "community structure microbial"[Title/Abstract] OR "Microbial Community Structures"[Title/Abstract] OR "Flora"[Title/Abstract] OR "Microflora"[Title/Abstract] OR "Dysbiosis"[Title/Abstract] OR "Ecosystem"[Title/Abstract] OR "Bacteria"[Title/Abstract] OR "gut-lung axis"[Title/Abstract] | 869065 |
| #3  "Medicine, Chinese traditional"[MeSH Terms] OR "Herbal Medicine"[MeSH Terms] OR "Medicine, Traditional"[MeSH Terms] OR "Herbal Medicine"[Title/Abstract] OR "Medicine, Herbal"[Title/Abstract] OR "Medicine, Chinese Traditional"[Title/Abstract] OR "Traditional Chinese Medicine"[Title/Abstract] OR "Traditional Medicine, Chinese"[Title/Abstract] OR "Zhong Yi Xue"[Title/Abstract] OR "Chinese Traditional Medicine"[Title/Abstract] OR "Chinese Medicine, Traditional"[Title/Abstract] OR "decoction"[Title/Abstract] OR "Ointments"[MeSH Terms] OR "Ointment"[Title/Abstract] OR "Salve"[Title/Abstract] OR "Salves"[Title/Abstract] OR "Unguents"[Title/Abstract] OR "Unguent"[Title/Abstract] OR "Pastes"[Title/Abstract] OR "Paste"[Title/Abstract] OR "bolus"[Title/Abstract] OR "Acupuncture"[MeSH Terms] OR "Acupuncture Therapy"[MeSH Terms] OR "Pharmacopuncture"[Title/Abstract] OR "Acupuncture Treatment"[Title/Abstract] OR "Acupuncture Treatments"[Title/Abstract] OR "Treatment, Acupuncture"[Title/Abstract] OR "Therapy, Acupuncture"[Title/Abstract] OR "Pharmacoacupuncture Treatment"[Title/Abstract] OR "Treatment, Pharmacoacupuncture"[Title/Abstract] OR" Pharmacoacupuncture Therapy"[Title/Abstract] OR "Therapy, Pharmacoacupuncture"[Title/Abstract] OR "Acupotomy"[Title/Abstract] OR "Acupotomies"[Title/Abstract] OR "Moxibustion"[MeSH Terms] OR "Moxibustion"[Title/Abstract] OR "Massage"[MeSH Terms] OR "Zone Therapy"[Title/Abstract] OR "Therapies, Zone"[Title/Abstract] OR "Zone Therapies"[Title/Abstract] OR "Therapy, Zone"[Title/Abstract] OR "Massage Therapy"[Title/Abstract] OR "Massage Therapies"[Title/Abstract] OR "Therapies, Massage"[Title/Abstract] OR "Therapy, Massage"[Title/Abstract] | 246129 |
| #4：#1 AND #2 AND 3#  ("asthma"[MeSH Terms] OR "asthma"[Title/Abstract] OR "asthmas"[Title/Abstract] OR "asthma bronchial"[Title/Abstract] OR "bronchial asthma"[Title/Abstract] OR "pulmonary disease, chronic obstructive"[MeSH Terms] OR "chronic obstructive pulmonary diseases"[Title/Abstract] OR "COPD"[Title/Abstract] OR "chronic obstructive lung disease"[Title/Abstract] OR "chronic obstructive pulmonary disease"[Title/Abstract] OR "COAD"[Title/Abstract] OR "chronic obstructive airway disease"[Title/Abstract] OR "airflow obstruction chronic"[Title/Abstract] OR "airflow obstructions chronic"[Title/Abstract] OR "chronic airflow obstructions"[Title/Abstract] OR "chronic airflow obstruction"[Title/Abstract] OR "chronic inflammatory airway disease"[Title/Abstract] OR "chronic inflammatory airway diseases"[Title/Abstract]) AND ("Microbiota"[MeSH Terms] OR "Microbiota"[Title/Abstract] OR "Microbial Community"[Title/Abstract] OR "Microbiotas"[Title/Abstract] OR "community microbial"[Title/Abstract] OR "Microbial Communities"[Title/Abstract] OR "Microbial Community Composition"[Title/Abstract] OR "community composition microbial"[Title/Abstract] OR "composition microbial community"[Title/Abstract] OR "Microbial Community Compositions"[Title/Abstract] OR "Microbiome"[Title/Abstract] OR "Microbiomes"[Title/Abstract] OR "Microbial Community Structure"[Title/Abstract] OR "community structure microbial"[Title/Abstract] OR "Microbial Community Structures"[Title/Abstract] OR "Flora"[Title/Abstract] OR "Microflora"[Title/Abstract] OR "Dysbiosis"[Title/Abstract] OR "Ecosystem"[Title/Abstract] OR "Bacteria"[Title/Abstract] OR "gut-lung axis"[Title/Abstract]) AND ("medicine, chinese traditional"[MeSH Terms] OR "Herbal Medicine"[MeSH Terms] OR "medicine, traditional"[MeSH Terms] OR "Herbal Medicine"[Title/Abstract] OR "medicine herbal"[Title/Abstract] OR "medicine chinese traditional"[Title/Abstract] OR "Traditional Chinese Medicine"[Title/Abstract] OR "traditional medicine chinese"[Title/Abstract] OR "Zhong Yi Xue"[Title/Abstract] OR "Chinese Traditional Medicine"[Title/Abstract] OR "chinese medicine traditional"[Title/Abstract] OR "decoction"[Title/Abstract] OR "Ointments"[MeSH Terms] OR "Ointment"[Title/Abstract] OR "Salve"[Title/Abstract] OR "Salves"[Title/Abstract] OR "Unguents"[Title/Abstract] OR "Unguent"[Title/Abstract] OR "Pastes"[Title/Abstract] OR "Paste"[Title/Abstract] OR "bolus"[Title/Abstract] OR "Acupuncture"[MeSH Terms] OR "Acupuncture Therapy"[MeSH Terms] OR "Pharmacopuncture"[Title/Abstract] OR "Acupuncture Treatment"[Title/Abstract] OR "Acupuncture Treatments"[Title/Abstract] OR "treatment acupuncture"[Title/Abstract] OR "therapy acupuncture"[Title/Abstract] OR "Pharmacoacupuncture Treatment"[Title/Abstract] OR "Pharmacoacupuncture Therapy"[Title/Abstract] OR "Acupotomy"[Title/Abstract] OR "Acupotomies"[Title/Abstract] OR "Moxibustion"[MeSH Terms] OR "Moxibustion"[Title/Abstract] OR "Massage"[MeSH Terms] OR "Zone Therapy"[Title/Abstract] OR "Zone Therapies"[Title/Abstract] OR "therapy zone"[Title/Abstract] OR "Massage Therapy"[Title/Abstract] OR "Massage Therapies"[Title/Abstract] OR "therapies massage"[Title/Abstract] OR "therapy massage"[Title/Abstract]) | 96 |
| Limits：Other Animals | 52 |
| Limits：English | 49 |
| Limits：2006.1.1-2026.1.5 | **47** |

1.2 **Web of Science search stratergy for 86**

| **Search** | **Hits** |
| --- | --- |
| 1#  TS=("Microbiota") OR AB=("Microbiota" OR "Microbial Community" OR "Microbial Communities" OR "Community, Microbial "OR "Microbial Community Composition*" OR "Community Composition, Microbial" OR "Composition, Microbial Community" OR "Microbial Community Structure*" OR "Community Structure, Microbial" OR "Microbiome*" OR "Flora" OR "Microflora" OR "Dysbiosis" OR "Ecosystem" OR "Bacteria" OR "gut-lung axis") | 1042503 |
| 2#  AB=("asthma*" OR "asthma, bronchial" OR "bronchial asthma" OR "chronic obstructive pulmonary disease*" OR "COPD" OR "pulmonary disease, chronic obstructive" OR "chronic obstructive lung disease" OR "COAD" OR "chronic obstructive airway disease*" OR "airflow obstruction*, chronic" OR "chronic airflow obstruction*" OR "chronic inflammatory airway disease*") | 182597 |
| 3#  TS=("Medicine, Chinese traditional" OR "Herbal Medicine" OR "Medicine, Traditional") OR AB=("Traditional Chinese Medicine" OR "Herbal Medicine" OR "decoction*" OR "Ointments*" OR "Salve*" OR "Unguent*" OR "Paste*" OR "bolus" OR "Acupuncture" OR "Acupuncture Therap*" OR "Acupuncture Treatment*" OR "Acupotomy" OR "Acupotomies" OR "Moxibustion" OR "Massage" OR "Zone Therap*" OR "Massage Therap*" ) | 216258 |
| 4#：1# AND 2# AND 3#  ((TS=("Microbiota") OR AB=("Microbiota" OR "Microbial Community" OR "Microbial Communities" OR "Community, Microbial "OR "Microbial Community Composition*" OR "Community Composition, Microbial" OR "Composition, Microbial Community" OR "Microbial Community Structure*" OR "Community Structure, Microbial" OR "Microbiome*" OR "Flora" OR "Microflora" OR "Dysbiosis" OR "Ecosystem" OR "Bacteria" OR "gut-lung axis")) AND (AB=("asthma*" OR "asthma, bronchial" OR "bronchial asthma" OR "chronic obstructive pulmonary disease*" OR "COPD" OR "pulmonary disease, chronic obstructive" OR "chronic obstructive lung disease" OR "COAD" OR "chronic obstructive airway disease*" OR "airflow obstruction*, chronic" OR "chronic airflow obstruction*" OR "chronic inflammatory airway disease*")) AND (TS=("Medicine, Chinese traditional" OR "Herbal Medicine" OR "Medicine, Traditional") OR AB=("Traditional Chinese Medicine" OR "Herbal Medicine" OR "decoction*" OR "Ointments*" OR "Salve*" OR "Unguent*" OR "Paste*" OR "bolus" OR "Acupuncture" OR "Acupuncture Therap*" OR "Acupuncture Treatment*" OR "Acupotomy" OR "Acupotomies" OR "Moxibustion" OR "Massage" OR "Zone Therap*" OR "Massage Therap*" ))) | 86 |
| Limits：English | 86 |
| Limits：2006-2026 | **86** |

1.3 **Embase search stratergy for 102**

| **Search** | **Hits** |
| --- | --- |
| #1  'microbiota*':ab,ti OR 'microbial community':ab,ti OR 'community, microbial':ab,ti OR 'microbial communities':ab,ti OR 'microbial community composition*':ab,ti OR 'community composition, microbial':ab,ti OR 'composition, microbial community':ab,ti OR 'microbial community structure*':ab,ti OR 'community structure, microbial':ab,ti OR 'microbiome*':ab,ti OR flora:ab,ti OR microflora:ab,ti OR dysbiosis:ab,ti OR ecosystem:ab,ti OR bacteria:ab,ti OR 'gut-lung axis':ab,ti | 968514 |
| #2  'asthma*':ab,ti OR 'asthma, bronchial':ab,ti OR 'bronchial asthma':ab,ti OR 'chronic obstructive pulmonary disease*':ab,ti OR 'COPD':ab,ti OR 'pulmonary disease, chronic obstructive':ab,ti OR 'chronic obstructive lung disease':ab,ti OR 'COAD':ab,ti OR 'chronic obstructive airway disease*':ab,ti OR 'airflow obstruction*, chronic':ab,ti OR 'chronic airflow obstruction*':ab,ti OR 'chronic inflammatory airway disease*':ab,ti | 464192 |
| #3  'Medicine, Chinese traditional':ab,ti OR 'Herbal Medicine':ab,ti OR 'Medicine, Traditional':ab,ti OR 'Traditional Chinese Medicine':ab,ti OR 'decoction*':ab,ti OR 'Ointments*':ab,ti OR 'Salve*':ab,ti OR 'Unguent*':ab,ti OR 'Paste*':ab,ti OR 'bolus':ab,ti OR 'Acupuncture':ab,ti OR 'Acupuncture Therap*':ab,ti OR 'Acupuncture Treatment*':ab,ti OR 'Acupotom*':ab,ti OR 'Moxibustion':ab,ti OR 'Massage':ab,ti OR 'Zone Therap*':ab,ti OR 'Massage Therap*':ab,ti | 310109 |
| 4#  1# AND 2# AND 3# | 151 |
| 5#  4# AND ('animal experiment'/de OR 'animal model'/de OR 'animal tissue'/de OR 'nonhuman'/de) AND [2006-2026]/py | **102** |

1.4 **Cochrane search stratergy for 57**

| **Search** | **Hits** |
| --- | --- |
| #1 MeSH descriptor: [Microbiota] explode all trees | 2924 |
| #2 ("Microbiota" OR "Microbial Community" OR "Microbial Communities" OR "Community, Microbial" OR "Microbial Community Composition" OR "Community Composition, Microbial" OR "Composition, Microbial Community" OR "Microbial Community Structure" OR "Community Structure, Microbial" OR "Microbiome" OR "Flora" OR "Microflora" OR "Dysbiosis" OR "Ecosystem" OR "Bacteria" OR "gut-lung axis"):ti,ab,kw (Word variations have been searched) | 34269 |
| #3 #1 or #2 | 34272 |
| #4 MeSH descriptor: [Asthma] explode all trees |  |
| #5 MeSH descriptor: [pulmonary disease, chronic obstructive] explode all trees |  |
| #6 ("asthma" OR "asthma, bronchial" OR "bronchial asthma" OR "chronic obstructive pulmonary disease" OR "chronic obstructive pulmonary diseases" OR "COPD" OR "chronic obstructive lung disease" OR "COAD" OR "chronic obstructive airway disease" OR "airflow obstruction, chronic" OR "airflow obstructions, chronic" OR "chronic airflow obstructions" OR "chronic airflow obstruction" OR "chronic inflammatory airway disease" OR "chronic inflammatory airway diseases"):ti,ab,kw (Word variations have been searched) | 61041 |
| #7 #4 or #5 or #6 | 61788 |
| #8 MeSH descriptor: [Medicine, Chinese traditional] explode all trees | 1932 |
| #9 MeSH descriptor: [Herbal Medicine] explode all trees | 95 |
| #10 MeSH descriptor: [Medicine, Traditional] explode all trees |  |
| #11 ("Medicine, Chinese traditional" OR "Herbal Medicine" OR "Medicine, Traditional" OR "Traditional Chinese Medicine" OR "decoction" OR "Ointment" OR "Salve" OR "Unguent" OR "Paste" OR "bolus" OR "Acupuncture" OR "Acupuncture Therapy" OR "Acupuncture Treatment" OR "Acupotomy" OR "Moxibustion" OR "Massage" OR "Zone Therapy" OR "Massage Therapy"):ti,ab,kw (Word variations have been searched) | 114275 |
| #12 #8 or #9 or #10 or #11 | 114626 |
| #6 #3 and #7 and #12 with Publication Year from 2006 to 2026 | **57** |

**Supplementary Table S3.** List of excluded studies following full-text review.

| **Title** | **Exclusion reason** |
| --- | --- |
| Phenotype-Specific Therapeutic Effect of Rhodiola wallichiana var. cholaensis Combined with Dexamethasone on Experimental Murine Asthma and Its Comprehensive Pharmacological Mechanism | Inappropriate interventions |
| Sodium houttuyfonate modulates the lung Th1/Th2 balance and gut microbiota to protect against pathological changes in lung of ovalbumin-induced asthmatic mice | Inappropriate interventions |
| Effects and mechanism of combination of Platycodon grandiflorum polysaccharides and Platycodon saponins in the treatment of chronic obstructive pulmonary disease rats through the gut-lung axis | Inappropriate interventions |
| Cordyceps militaris alleviates COPD by regulating amino acid metabolism, gut microbiota and short chain fatty acids | Inappropriate interventions |
| Lycium barbarum Polysaccharide Extracted from Lycium barbarum Leaves Ameliorates Asthma in Mice by Reducing Inflammation and Modulating Gut Microbiota | Inappropriate interventions |
| Theory of the exterior-interior relationship between the lungs and the large intestine to explore the mechanism of Eriobotrya japonica leaf water extract in the treatment of cough variant asthma | Inappropriate interventions |
| Ephedra sinica polysaccharide alleviates airway inflammations of mouse asthma-like induced by PM2.5 and ovalbumin via the regulation of gut microbiota and short chain fatty acid | Inappropriate interventions |
| Cordyceps militaris polysaccharide alleviates ovalbumin-induced allergic asthma through the Nrf2/HO-1 and NF-κB signaling pathways and regulates the gut microbiota | Inappropriate interventions |
| Mechanisms of Aletris spicata (Thunb.) Franch. Extract in Asthma Therapy: Oxidative Stress, Inflammation, and Gut Microbiota | Inappropriate interventions |
| Combination of Chinese and Western Medicine Optimizes the Intestinal Microbiota of Exacerbated Chronic Obstructive Pulmonary Disease in Rats | Inappropriate interventions |
| Resveratrol treatment alters lung microbiome in the murine ovalbumin-induced asthma model by increasing mucus-degrading Akkermansia muciniphila | Inappropriate interventions |
| Lycium barbarum polysaccharides improve gut microbiota composition and alleviate pulmonary inflammatory damage in allergic asthma mice by inhibiting the IL-15RA/FUT2 pathway | Inappropriate interventions |
| Astragalus polysaccharide alleviates asthma by modulating gut microbiota and serum metabolomics | Inappropriate interventions |
| Dietary Indian frankincense (Boswellia serrata) ameliorates murine allergic asthma through modulation of the gut microbiome | Inappropriate interventions |
| Ginger-processed Magnoliae Officinalis Cortex ameliorates ovalbumin-induced asthma by alleviating inflammation via the gut-lung axis | Inappropriate interventions |
| Amelioration of Inflammation in Rats with Experimentally Induced Asthma by Spenceria ramalana Trimen Polyphenols via the PI3K/Akt Signaling Pathway | Inappropriate interventions |
| Antibiotic-driven and microbiota-targeted therapy for advanced management of pulmonary hypertension | Inappropriate subjects |
| Effects of compound Caoshi silkworm granules on stable COPD patients and their relationship with gut microbiota: A randomized controlled trial | Inappropriate subjects |
| Epicatechin protects mice against OVA-induced asthma through inhibiting airway inflammation and modulating gut microbiota | Inappropriate interventions |
| Promising Intestinal Microbiota Associated with Clinical Characteristics of COPD Through Integrated Bioinformatics Analysis | No available data |

**Supplementary Table S4.** Full breakdown of study quality assessment for the included animal studies using the Systematic Review Center for Laboratory Animal Experimentation Risk of Bias tool.

| **Study ID** | **Selection bias** | | | **Performance bias** | | **Detection bias** | | **Attrition bias** | **Reporting bias** | **Others** | **Quality score** |
| --- | --- | --- | --- | --- | --- | --- | --- | --- | --- | --- | --- |
|  | **1** | **2** | **3** | **4** | **5** | **6** | **7** | **8** | **9** | **10** |  |
| **Miaomiao Di (2025)** | Unclear | Unclear | Unclear | Unclear | Unclear | Unclear | Unclear | High | Low | Low | 2 |
| **Yanfei Hong (2025)** | Unclear | Unclear | Unclear | Low | Unclear | Unclear | Unclear | Low | Low | Low | 4 |
| **Yushan Zhao (2025)** | Low | Unclear | Unclear | Low | Unclear | Unclear | Unclear | Low | Low | Low | 5 |
| **Yanlin Li (2025)** | Unclear | Unclear | Unclear | Low | Unclear | Unclear | Unclear | Low | Low | Low | 4 |
| **Wenting Xu (2025)** | Low | Unclear | Unclear | Low | Unclear | Unclear | Unclear | Low | Low | Low | 5 |
| **SiSi Han (2025)** | Unclear | Low | Low | Low | Low | Unclear | Unclear | Unclear | Low | Low | 6 |
| **Wei-Hsiang Hsu (2021)** | Unclear | Unclear | Unclear | Unclear | Unclear | Unclear | Unclear | High | Low | Low | 2 |
| **Yu Jia (2022)** | Unclear | Unclear | Unclear | Low | Unclear | Unclear | Unclear | Unclear | Low | Low | 3 |
| **Yanhua Kong (2016)** | Unclear | Low | Unclear | Low | Unclear | Unclear | Unclear | Unclear | Low | Low | 4 |
| **Yongan Wang (2021)** | Unclear | Unclear | Unclear | Low | Unclear | Unclear | Unclear | Unclear | Low | Low | 3 |
| **Yan Zhu (2020)** | Unclear | Unclear | Unclear | Low | Unclear | Unclear | Unclear | High | Low | Low | 3 |
| **Jing Mao (2022)** | Unclear | Unclear | Unclear | Low | Unclear | Unclear | Unclear | High | Low | Low | 3 |
| **Fei Liu (2023)** | Unclear | Unclear | Unclear | Unclear | Unclear | Unclear | Unclear | High | Low | Low | 2 |
| **JunMei Wang (2023)** | Unclear | Unclear | Unclear | Low | Unclear | Unclear | Unclear | Unclear | Low | Low | 3 |
| **Rui He (2023)** | Low | Unclear | Unclear | Low | Unclear | Unclear | Unclear | High | Low | Low | 4 |
| **Yuan Ruan (2023)** | Low | Unclear | Unclear | Low | Unclear | Unclear | Unclear | Low | Low | Low | 5 |
| **Lin Lin (2025)** | Unclear | Unclear | Unclear | Low | Unclear | Unclear | Unclear | High | Low | Low | 3 |
| **Yuanyuan He (2022)** | Unclear | Unclear | Unclear | Unclear | Unclear | Unclear | Unclear | Low | Low | Low | 3 |
| **Jiao Jiao (2022)** | Unclear | Low | Unclear | Low | Unclear | Unclear | Unclear | Low | Low | Low | 5 |
| **Haibo Hu (2023)** | Unclear | Unclear | Unclear | Low | Unclear | Unclear | Unclear | High | Low | Low | 3 |
| **Yuanyuan Li (2024)** | Unclear | Unclear | Unclear | Low | Unclear | Unclear | Unclear | High | Low | Low | 3 |
| **Yantong Yu (2025)** | Unclear | Unclear | Unclear | Unclear | Unclear | Unclear | Unclear | High | Low | Low | 2 |
| **Yanfei Hong (2025)** | Unclear | Unclear | Unclear | Low | Unclear | Unclear | Unclear | Low | Low | Low | 4 |
| **Lingmiao Wen (2023)** | Unclear | Unclear | Unclear | Low | Low | Unclear | Unclear | Low | Low | Low | 5 |
| **Yueying Wu (2022)** | Unclear | Unclear | Unclear | Unclear | Unclear | Unclear | Unclear | Unclear | Low | Low | 2 |

**Criteria for each item:** Item 1: sequence generation, item 2: baseline characteristics, item 3: Allocation concealment (selection bias); item 4: random housing, item 5: blinding (performance bias); item 6: random outcome assessment, item 7: blinding (detection bias); item 8: incomplete outcome data (attrition bias); item 9: selective outcome reporting (reporting bias). item 10: other sources of bias (other bias).

**Judgement:** The items judged as “Low” were scored 1 point, and both “High” and “Unclear” were scored as 0 point. The sum scores of the 10 items were presented as the quality score of each study.

**Supplementary Table S5.** Summary of beta diversity assessments in the included studies.

| Study | β-diversity | Findings |
| --- | --- | --- |
| Miaomiao Di (2025) | PCoA, ANOSIM | There was a clear separation between QHYFF and COPD groups (*P* = 0.009), while DXM treatment showed weak regulation effects (*P* = 0.099). |
| Yanfei Hong (2025) | PCoA based on Jaccard distances | There was a unremarkable difference between the MXSG and the model group (*P* = 0.338). |
| Wenting Xu (2025) | Hierarchical clustering based on Bray-Curtis and UPGMA | In BALF and fecal sample, there were differences in OVA and healthy group. Both LJZD and DXMS groups reduced the difference with healthy group to varying degrees. |
| Yu Jia (2022) | PCoA, PERMANOVA | PCoA showed distinctly different gut contents and BALF microbiota among healthy , model, and QBPF groups (*P* < 0.001). QBPF group was closer to the healthy group. |
| Yanhua Kong (2016) | PCoA based on Unweighted-UniFrac distances | The gut microbiota structure of treated groups significantly deviated from the baseline of the model group and had a smaller difference from the healthy group. |
| Yan Zhu (2020) | PCoA based on Unweighted-UniFrac distances | There was a significant separation between the healthy and AAI groups (*P* < 0.05) as well as between CP treatment and AAI groups (*P* < 0.01) and no significant separation between the CP and healthy group (*P* > 0.05). |
| Yongan Wang (2021) | PCoA based on Bray-Curtis and Jaccard distances, PERMANOVA | There was a significant separation between three groups (Bray-Curtis distance, *P* = 0.001; Jaccard distances, *P* = 0.001). |
| JunMei Wang (2023) | PCoA based on Weighted-Unifrac distance; NMDS based on Bray-Curtis distance | There were significant differences between the COPD and other groups. |
| Rui He (2023) | NMDS based on weighted unifrac distance | There was an obvious boundary line between the asthma modele and normal group, After treatment for 4 weeks, gut microbiota in SGT-treated asthma groups (M_MSGT and M_HSGT) was close to that of the normal group. |
| Yuan Ruan (2023) | PCoA based on Unifrac distance | There is a significant trend of separation between the normal and model groups, a significant trend of separation between the TD and model groups, and a significant trend of proximity between the TD and normal groups (*P* = 0.002). |
| Yuanyuan He (2022) | Weighted Unifrac clustering tree analysis; PCA; PCoA; NMDS | Gut microbial numbers and species differed significantly between the asthma model group and healthy group. Xiaoning group was similar to healthy group, while no significant difference was found between budesonide group and Xiaoning group. |
| Jiao Jiao (2022) | PCA; UPGMA | In gut microbiota, the control and model groups were basically separated, and the drug group clustered closer to the control group. In lung microbiota, the three groups were largely clustered within their respective groups, although the drug group showed a trend toward the control group. |
| Haibo Hu (2023) | PCoA and clustering analysis | Model group were completely separated from the control group, and HD-QFSS group were closer to control group. |
| Yuanyuan Li (2024) | Cluster tree analysis; PCA; NMDS | Bacterial structure of the control group was separated from that of the model group and the BFHX administration group was similar to that of the model group. BFHX could improve bacterial percentage. |
| Yanfei Hong (2025) | PCoA, PERMANOVA | There was a significant separation of overall community structures in TMDC-treated mice compared to asthmatic mice across all gut segments. |

Note: PCoA: Principal Coordinate Analysis; ANOSIM: Analysis of Similarities; UPGMA: Unweighted Pair Group Method with Arithmetic Mean; PERMANOVA: Permutational Multivariate Analysis of Variance; NMDS: Non-metric Multidimensional Scaling; PCA: Principal Component Analysis.

**Supplementary Table S6.** Summary of differences in microbial taxa abundance in the included studies.

| **Study** | **Site** | **Findings** |
| --- | --- | --- |
| Miaomiao Di (2025) | Intestine | At the phylum level, QHYFF effectively enriched *Actinobacteria* and *Bacteroidetes* (*P* < 0.05), and DXM enriched *Proteobacteria* (*p* < 0.05). QHYFF and DXM significantly decreased the abundance of *Firmicutes* (*P* < 0.05, *P* < 0.01). At the genus level, both QHYFF and DXM enriched *Parasutterella* (*P* < 0.05, *P* < 0.01). Furthermore, QHYFF significantly increased the relative abundance of *Bifidobacterium*, *Faecalibaculum*, *Blautia*, *Butyricimonas*, *Allobaculum* and *Ruminococcus* torques group (*P* < 0.05), and markedly reduced the relative abundance of *Helicobacter* (*P* < 0.01) and *Lactobacillus* (*P* < 0.05). |
| Yanfei Hong (2025) | Intestine | At the genus level, MXSG significantly reduced the abundances of *Pseudomonas_E*, *Gemmiger_A*, and *Apilactobacillus*. MXSG prominently promoted the abundance of potential probiotics including *Lactococcus_A*, *Lactobacillus*, *Limosilactobacillus*, and *Turicibacter*. at the species level, MXSG increased the abundance of *Paraprevotella* *clara*, *Lactobacillus_intestinalis*, and *Bacteroides_H faecalis* in asthmatic mice. |
| Yushan Zhao (2025) | Intestine | Compared to the model group, the abundance of *Lactobacillus*, *norank_f_Muribaculaceae*, *Clostridium_sensu_stricto_1*, *Blautia* and other bacterial genera was upregulated in the TP-TR group. The abundances of *Romboutsia*, n*orank_f_norank_o_Clostridia_UCG-014*, *UCG-005*, *Ruminococcus*, *turicibacter*, *Aerococcus* and other genera decreased. |
| Yanlin Li (2025) | Intestine | At the phylum level, the OVA group exhibited significantly higher abundance of Bacteroidetes phylum compared to the Firmicutes phylum which was lower than JEQS and vehicle groups. At the genus level, there were significant differences in abundance rankings among samples from different groups, with the highest abundance in each group being Muribaculaceae genus, followed by Clostridium genus, Lactobacillus genus, and Bacteroides genus for the control group, and Actinobacillus genus, Lactobacillus genus, Prevotella genus, Pseudoprevotella genus, and Barnesiella genus for the OVA group. Finally, the JEQS group exhibited abundance rankings of Actinobacillus genus, Lactobacillus genus, Clostridium genus, Escherichia genus, Parasutterella genus, and Sutterella genus. |
| SiSi Han (2025) | Airway; Intestine | There were 70 identical floral species out of a total of 162 species detected in the lungs and 84 species detected in the intestines. , there was a marked decrease in the abundance of Alloprevotella spp. and Roseburia spp. in both the lungs and intestines of the model group (*P* < 0.05), while Oscillibacter spp. Decreased only in the intestines (*P* < 0.05).YQXF partially restored bacterial abundance in the lungs and intestines. |
| Wei-Hsiang Hsu (2021) | Intestine | The ratio of *Firmicutes* to *Bacteroidetes* abundance in control, *Derp*, and *Derp*+YGW groups was 0.59, 11.49, and 0.70, respectively. Derp significantly enhanced the ratio, and YGW treatment reversed the ratio, but without significant difference between the control and *Derp*+YGW group. |
| Yu Jia (2022) | Airway; Intestine | For gut microbiota, The dominant phyla in the three groups, accounting for more than 97%, include *Bacteroidetes*, *Firmicutes*, *Proteobacteria*, and *Fusobacteria*.The relative abundance of *Fusobacteria* was decreased in the model group than in the control group and increased after QBPF intervention. Regarding pulmonary microbiota, the top five dominant phyla were *Bacteroidetes*, *Proteobacteria*, *Firmicutes*, *Tenericutes*, and A*ctinobacteria*, which accounted for over 92% of the total sequences in all three groups. Compared to the control group, the proportion of *Bacteroidetes*, *Proteobacteria*, *Firmicutes*, and *Actinobacteria* declined in the model group and climbed in the QBPF group, and the *Tenericutes* level rose in the model group and dropped in the QBPF group. |
| Yanhua Kong (2016) | Intestine | Compared with the model group, the relative abundance of *Prevotella* and *Lactobacillus* in the control group was higher, while that of *Bacteroides*, *Parabacteroides*, *Paraprevotella*, *Roseburia*, *Ruminococcus*, *Faecalibacterium, Allobaculum, Sutterella, Desulfovibri*o, *Erwinia*, and *Escherichia* was lower. *Adlercreutzia* was moreprevalent in the RLD groups (*P* = 0.036) and *Erysipelotrichaceae* was more prevalent in the DEX group (*P* = 0.038). Compared with the model group, the relative abundance of *Lactobacillus* was clearly increased in the DEX group, while the relative abundance of *Escherichia* was markedly increased in the RLD groups. |
| Yongan Wang (2021) | Intestine | *Gordonibacter*, *Allobaculum*, *Tyzzerela_3*, *Akkermansia*, and *Subdoligranulum* were less abundant in COPD mice than controls, whereas they were significantly enriched upon the M-XBCQ administration. *Streptococcus*, *Marvinbryantia*, *Candidatus_Stoquefichus*, and *Coriobacteriaceae_UCG-002* exhibited higher relative proportions in the colon of COPD mice compared to the control group. However, the M-XBCQ treatment remarkably inhibited the accumulation of *Streptococcus* and *Marvinbryantia*. |
| Yan Zhu (2020) | Intestine | At the phylum level, gut microbiota was mainly composed of *Firmicutes*, *Bacteroidetes*, *Proteobacteria*, *Actinobacteria*, and *Saccharibacteria* among the groups. In the AAI group, the relative abundance of B*acteroidetes*, *Proteobacteria*, and *Saccharibacteria* significantly decreased (*P* < 0.05, *P* < 0.01, *P* < 0.01) and the relative abundance of *Firmicutes* increased (*P* < 0.01) compared with the CN group. Compared with the AAI group, the CP group showed significantly decreased abundance of *Firmicutes* (*P* < 0.05) and increased abundance of *Bacteroidetes*, *Proteobacteria*, and *Saccharibacteria* (*P* < 0.01, *P* < 0.01, *P* < 0.01). |
| Jing Mao (2022) | Intestine | 90-97% of the gut bacterial community was composed of *Firmicutes* (91.50%), *Bacteroides* (3.02%), *Proteobacteria* (1.46%), *Spirochaetes* (1.31%), and *TM7* (1.28%) in normal rats. A decreased proportion of *Firmicutes* and increased proportions of *Bacteroides*, *Proteobacteria* and *Spirochaetes* were observed in COPD rats. |
| Fei Liu (2023) | Intestine | The top three bacteria in the phylum were *Firmicutes*, *Bacteroidetes*, and *Proteobacteria*, whereas the top genera were *Clostridium_XIVa*, *Alistipes*, and *Oscillibacter*. At the family level, members of *Ruminococcaceae* and *Lachnospiraceae* were reduced by Pingchuan formula (PC) treatment. At the genus level, PC treatment increased the relative abundance of *Clostridium_IV*, *Clostridium_XIVa*, *Akkmansia* and *Roseburia*, while decreased *Blautia*, *Barnesiella* and *Clostridium_III* compared with the model group. At the species level, *Clostridium_methylpentosum* and *Clostridium_aldenenie* were up-regulated while *Clostridium_viride* and *Barnesiella_intestinhominis* were down-regulated by PC treatment. |
| JunMei Wang (2023) | Intestine | At the phylum level, COPD group showed decreased abundances of *Firmicutes* and *Proteobacteria*, and increased abundances of *Bacteroidetes* and *Tenericutes* compared with the control group. There was callback trend of *Bacteroidetes* and *Firmicutes* in SWP and DEX groups. At the family level, COPD significantly decreased the abundances of *Aerococcus*, *Lactobacillus*, and *Psychrobacter* compared with the control group, and SWP and DEX treatment reversed these changes. |
| Rui He (2023) | Intestine | At the phylum level, M_MSGT group displayed higher abundance of *Patescibacteria* (*P* < 0.05) compared with the Model group. At the genus level, vs. the Normal group, the Model group displayed a higher abundance of *Turicibacter* (*P* < 0.05) and *Ruminococcaceae_UCG-010* (*P* < 0.05), and lower abundance of *Ruminococcaceae_UCG-014* (*P* < 0.05). There was a significant difference of the microbial community in the gut microbiota between the Normal and Model group, and the gut microbiota structure changed and recovered after SGT intervention. |
| Yuan Ruan (2023) | Intestine | Tingli Dazao Xiefei Decoction group (TD) has a significant modification effect on the intestinal flora of asthmatic rats. At the genus level, rats in the normal group shared changes with the model group and rats in the model group with the TD group in the *g_Lachnospiraceae_NK4A136*, *g_Prevotellaceae_Ga6A1*, *g_UCG_005*, *g_Intestinimonas* and *g_Colidextribacter*. |
| Lin Lin (2025) | Intestine | OVA exposure induced no significant changes in the relative abundances of the major phyla (all *P* > 0.05), but significantly decreased the abundance of class *Clostridia* and increased that of class *Bacilli* (both *P* < 0.001), while it elevated the abundances of family *Lactobacillaceae* and genus *Lactobacillus* (both *P* < 0.001). |
| Yuanyuan He (2022) | Intestine | The abundance values of *Staphylococcus* in the healthy group were significantly higher than those in the budesonide and Xiaoning groups, but it was opposite to *Clostridiales*. *Mucispirillum* had a lower and *Parasutterella* had a higher abundance value in the XN group than in the other three groups. The abundance value of *Corynebacterium* in the XN group significantly decreased compared with the control group, so did Helicobacter compared with the budesonide group. These results indicated that Xiaoning liquid changed the community structure of gut bacteria and inhibited the proliferation of some. |
| Jiao Jiao (2022) | Airway; Intestine | There was a significant difference in gut microbiota abundance among control, model and XBCQD groups at the phylum, class, order, family, and genus levels. The XBCQD group showed increased microbial abundance. For lung microbiota, significant differences were only observed at the genus level. Compared with the model group, XBCQD showed a tendency to improve *Pseudomonas*, *Microbacterium*, and *Allobaculum*. |
| Haibo Hu (2023) | Intestine | At the phylum level, *Firmicutes*/*Bacteroidetes* ratio was significantly higher in the model group than in the control group (*P* < 0.01), and signifcantly lower in the HD-QFSS group than in the model group. At the genus level, asthmatic model group showed significantly higher relative abundances of *Lachnospiraceae_NK4A136_group* and *Helicobacter* (both *P* < 0.05), and significantly lower abundances of *Lactobacillus*, *Alloprevotella*, *Bacteroidota*, and *Dubosiella* (both *P* < 0.05) compared with control group. QFSS signifcantly increased the relative abundances of *Lactobacillus* (*P* < 0.01) and *Dubosiella* (*P* < 0.05), and decreased the those of *Lachnospiraceae_NK4A136_group* and *Helicobacter* (both *P* < 0.05) compared with the model group. |
| Yuanyuan Li (2024) | Intestine | At the phylum level, the proportion of *Verrucomicrobiota* and *Desulfobacterota* increased while that of *Actinobacteria* decreased in the MOD group compared with the CTL group. BFHX reversed the proportion of bacteria at the phylum level. At the genus level, the proportions of *Murbaculaceae*, *Lactobacillus*, *Desulfuribrio*, *Clostridiumthe*, and *Akkermansia* in the CTL group decreased sharply compared to those in the MOD group, whereas the proportions of *Lactobacillus*, *Desulfuribrio*, and *Bacteroidetes* increased substantially, and BFHX reversed the proportions. |
| Yantong Yu (2025) | Intestine | The control group was enriched with *Romboutsia*, *Ligilactobacillus*, and *Lactobacillus*. *Streptococcus* was more abundant in the model group. Compared with the model group, the control group showed higher levels of *Dubosiella*, *Candidatus*_ *Arthromitus*, *Romboutsia*, and *Rothia*. *Streptococcus* significantly increased after modeling (*P* < 0.05) and significantly decreased after treatment (*P* < 0.05). |
| Yanfei Hong (2025) | Intestine | TMDC treatment increased the relative abundance of *Firmicutes* and *Proteobacteria* while decreasing that of *Bacteroidetes*, which are the three dominant phyla across the gut segments. Mice in the TMDC treatment group exhibited higher proportions of the genus *Lactobacillus*, particularly in the small intestine and colon. |

Supplementary Table S7. Alpha-diversity data used in the quantitative synthesis of intestinal microbiota.

| Alpha-diversity index | Study | Site | Sample | Subgroup | Number of animals | | | Mean ± SD | | |
| --- | --- | --- | --- | --- | --- | --- | --- | --- | --- | --- |
|  |  |  |  |  | Model group | TCM group | Control group | Model group | TCM group | Control group |
| Observed-species | Yanlin Li (2025) | Intestine | Fecal | BA | 6 | 6 | 6 | 2234.30±58.27 | 1777.18±381.37 |  |
|  | Yan Zhu (2020) | Intestine | Fecal | BA | 5 | 5 | 5 | 259.17±113.61 | 497.04±24.85 |  |
|  | JunMei Wang (2023) | Intestine | Fecal | COPD | 10 | 30 | 10 | 745.99±265.45 | 1048.65±141.77 | 998.76±214.23 |
|  | Lin Lin (2025) | Intestine | Fecal | BA | 5 | 5 | 5 | 854.05±118.92 | 972.97±86.49 | 908.11±86.49 |
|  | Jiao Jiao (2022) | Intestine | Fecal | COPD | 8 | 8 | 8 | 776.50±92.31 | 941.17±187.54 |  |
|  | Yantong Yu (2025) | Intestine | Fecal | BA | 6 | 12 | 6 | 543.16±300.89 | 701.84 ± 126.98 | 735.90±142.08 |
| Chao1 | Yanfei Hong (2025) | Intestine | Fecal | BA | 6 | 6 | 6 | 827.21+125.98 | 966.88+144.07 |  |
|  | Yushan Zhao (2025) | Intestine | Fecal | COPD | 6 | 6 | 6 | 931.58+121.05 | 915.79+52.63 | 815.79+131.58 |
|  | WenTing Xu (2025) | Intestine | Fecal | BA | 6 | 6 | 6 | 789.58+127.35 | 322.72+45.71 | 303.72+16.39 |
|  | Yanlin Li (2025) | Intestine | Fecal | BA | 6 | 6 | 6 | 2735.29+66.46 | 2414.13+410.32 |  |
|  | Yu Jia (2022) | Intestine | Fecal | COPD | 8 | 8 | 8 | 4475.54+337.98 | 4476.1+85.46 |  |
|  | Yan Zhu (2020) | Intestine | Fecal | BA | 5 | 5 | 5 | 345.56+123.08 | 568.05+28.4 |  |
|  | Jing Mao (2022) | Intestine | Fecal | COPD | 12 | 12 | 12 | 2625.17+78.74 | 3214.14+173.19 | 2842.49+94.49 |
|  | Fei Liu (2023) | Intestine | Fecal | BA | 8 | 8 | 8 | 379.99+12.17 | 384.8+23.79 | 389.02+16.32 |
|  | JunMei Wang (2023) | Intestine | Fecal | COPD | 10 | 30 | 10 | 1045.85+192.33 | 1236.04 ± 123.59 | 1189.17+330.43 |
|  | Yuan Ruan (2023) | Intestine | Fecal | BA | 8 | 8 | 8 | 329.35+76.96 | 556.96+67.01 |  |
|  | Yuanyuan He (2022) | Intestine | Fecal | BA | 15 | 15 | 15 | 635.72+22.08 | 613.63+16.36 | 626.8+46.62 |
|  | Jiao Jiao (2022) | Intestine | Fecal | COPD | 8 | 8 | 8 | 880.46+95.97 | 1030.63+162.03 |  |
|  | Yantong Yu (2025) | Intestine | Fecal | BA | 6 | 12 | 6 | 587.67+294.2 | 705.44 ± 214.23 | 798.8+83.58 |
| Shannon | Yanfei Hong (2025) | Intestine | Fecal | BA | 6 | 6 | 6 | 5.60±0.62 |  |  |
|  | Yushan Zhao (2025) | Intestine | Fecal | COPD | 6 | 6 | 6 | 3.68±0.48 | 3.57±0.14 |  |
|  | WenTing Xu (2025) | Intestine | Fecal | BA | 6 | 6 | 6 | 8.14±0.15 | 6.92±0.40 | 6.82±0.12 |
|  | Yanlin Li (2025) | Intestine | Fecal | BA | 6 | 6 | 6 | 7.99±0.10 | 6.64±1.24 |  |
|  | WeiHsiang Hsu (2021) | Intestine | Fecal | BA | 4 | 4 | 4 | 2.80±2.68 | 5.14±1.80 |  |
|  | Yan Zhu (2020) | Intestine | Fecal | BA | 5 | 5 | 5 | 3.29±1.00 | 6.19±0.52 |  |
|  | Jing Mao (2022) | Intestine | Fecal | COPD | 12 | 12 | 12 | 7.46±0.31 | 8.07±0.41 |  |
|  | Fei Liu (2023) | Intestine | Fecal | BA | 8 | 8 | 8 | 4.03±0.17 | 4.28±0.26 | 4.11±0.19 |
|  | JunMei Wang (2023) | Intestine | Fecal | COPD | 10 | 30 | 10 | 3.31±0.69 | 6.09 ± 0.86 | 5.09±1.87 |
|  | Rui He (2023) | Intestine | Fecal | BA | 9 | 9 | 9 | 6.47±0.27 |  |  |
|  | Lin Lin (2025) | Intestine | Fecal | BA | 5 | 5 | 5 | 5.49±0.78 |  | 5.73±0.54 |
|  | Yuanyuan He (2022) | Intestine | Fecal | BA | 15 | 15 | 15 | 4.64±0.02 | 4.70±0.01 | 4.69±0.01 |
|  | Jiao Jiao (2022) | Intestine | Fecal | COPD | 8 | 8 | 8 | 4.62±1.20 | 5.93±0.63 |  |
|  | Haibo Hu (2023) | Intestine | Fecal | BA | 6 | 6 | 6 | 5.77±0.38 | 5.80±0.51 |  |
|  | Yantong Yu (2025) | Intestine | Fecal | BA | 6 | 12 | 6 | 3.93±2.09 | 4.90 ± 1.40 | 5.61±0.90 |
|  | Yanfei Hong (2025) | Jejunum | Fecal | BA | 6 | 6 | 6 | 3.40±0.61 | 2.15±1.39 | 1.90±2.79 |
|  |  | Ileum |  |  | 6 | 6 | 6 | 3.04±1.50 | 1.70±1.24 | 1.27±0.89 |
|  |  | Cecum |  |  | 6 | 6 | 6 | 5.53±0.94 | 4.83±0.61 | 5.27±1.25 |
|  |  | Colon |  |  | 6 | 6 | 6 | 5.14±0.71 | 5.16±0.31 | 4.47±0.55 |

Note: L: Low-dose group, M: Medium-dose group, H: High-dose group. For JunMei Wang (2023), the L, M, and H TCM arms (n = 10 per arm) were combined before meta-analysis. For Yantong Yu (2025), the L and H TCM arms (n = 6 per arm) were combined before meta-analysis. The shared comparator group was entered only once within each outcome-specific comparison. Simpson-derived measures were not included in the quantitative synthesis because their reported definitions and directions were not consistently verifiable across studies.

Supplementary Table S8. Alpha-diversity data used in the quantitative synthesis of airway microbiota.

| Alpha-diversity index | Study | Site | Sample | Subgroup | Number of animals | | | Mean ± SD | | |
| --- | --- | --- | --- | --- | --- | --- | --- | --- | --- | --- |
|  |  |  |  |  | Model group | TCM group | Control group | Model group | TCM group | Control group |
| Chao1 | WenTing Xu (2025) | Airway | BALF | BA | 6 | 6 | 6 | 402.93±46.27 | 248.5±41.02 | 248.52±28.39 |
|  | Yu Jia (2022) | Airway | BALF | COPD | 8 | 8 |  | 2528.14±1141.55 | 2998.15±191.55 |  |
|  | Lingmiao Wen (2023) | Airway | BALF | BA | 8 | 24 | 8 | 539.49±77.70 | 328.00 ± 127.56 | 223.15±108.77 |
|  | Jiao Jiao (2022) | Airway | BALF | COPD | 8 | 8 |  | 242.88±21.41 | 227.58±43.29 |  |
|  | Yueying Wu (2022) | Airway | lung tissuel | COPD | 5 | 5 |  | 1406.02±1923.62 | 1269.92±899.54 |  |
| Shannon | WenTing Xu (2025) | Airway | BALF | BA | 6 | 6 | 6 | 7.09±0.14 | 5.79±0.38 | 6.05±0.35 |
|  | Lingmiao Wen (2023) | Airway | BALF | BA | 8 | 24 | 8 | 3.34±0.48 | 2.80 ± 0.51 | 2.61±0.46 |
|  | Jiao Jiao (2022) | Airway | BALF | COPD | 8 | 8 |  | 2.10±0.34 | 2.47±0.47 |  |
|  | Yueying Wu (2022) | Airway | lung tissuel | COPD | 5 | 5 |  | 1.87±1.16 | 5.02±0.79 |  |

Note: L: Low-dose group, M: Medium-dose group, H: High-dose group. For Lingmiao Wen (2023), the L, M, and H TCM arms (n = 8 per arm) were combined before meta-analysis. The shared comparator group was entered only once within each outcome-specific comparison.
